# Iron‐Tannic Acid Hydrogel Mediated Mild Photothermal Therapy Combined With Ferroptosis Enhances Anti‐Tumor Immunity in Colorectal Cancer

**DOI:** 10.1002/advs.77186

**Published:** 2026-08-25

**Authors:** Yuan Zhu, Xi Yu, Tao Wang, Wanyi Liu, Chang Lu, Jiani Zhong, Yujie He, Wei Li, Kunxi Zhang, Peihao Yin, Zeting Yuan

**Affiliations:** ^1^ Central Laboratory Putuo Hospital Shanghai University of Traditional Chinese Medicine Shanghai China; ^2^ Department of General Surgery Putuo Hospital Shanghai University of Traditional Chinese Medicine Shanghai China; ^3^ Department of Polymer Materials School of Materials Science and Engineering Shanghai University Shanghai China; ^4^ Shanghai Putuo Central School of Clinical Medicine Anhui Medical University Shanghai China; ^5^ Shanghai Putuo People's Hospital Affiliated to Tongji University Shanghai China

**Keywords:** colorectal cancer, ferroptosis, immunotherapy, iron‐tannic acid hydrogel, mild photothermal therapy

## Abstract

Postoperative recurrence and metastasis are the primary causes of mortality in colorectal cancer (CRC). And its immunosuppressive tumor microenvironment (TME) often results in the poor response to immune checkpoint blockade (ICB). This study designed an injectable and adhesive tannic acid‐iron hydrogel for locally mild photothermal therapy (PTT, ≤ 45°C) and ferroptosis to prevent the postoperative recurrence and metastasis in combination with programmed death ligand 1 (PD‐L1) checkpoint blockade immunotherapy. First, this system was assembled from tannic acid (TA) and gelatin microspheres to form an injectable and adhesive hydrogel (Gel/TA). Subsequently, the metal‐polyphenol network (MPNs) was introduced via Fe^3+^‐TA interactions, providing photothermal conversion and Fe^3+^ release capabilities (Gel/TA/Fe^3+^). Gel/TA/Fe^3+^ hydrogels not only ablated tumor cells and induced immunogenic cell death (ICD) under near‐infrared (NIR) irradiation, but also elicited ferroptosis of both tumor cells and tumor‐associated macrophage (TAMs), resulting in dendritic cells (DCs) maturation, T‐cell infiltration and down‐regulation of ferroptosis‐sensitive M2‐type TAMs levels. Therefore, in cooperation with αPD‐L1, Gel/TA/Fe^3+^ could not only evoke an immune response against the primary tumor, but also promote an abscopal effect against distant metastatic tumor. This work presented a novel material for CRC postoperative therapy via the synergistic effect of immediate tumor‐cell killing and enhanced immunotherapy.

## Graphical abstract

An injectable gelatin‐based tannic acid–iron hydrogel with mild photothermal therapy could not only induce immunogenic cell death, but also elicit ferroptosis of both tumor cells and tumor‐associated macrophages, resulting in DC maturation, T‐cell infiltration and down‐regulation of ferroptosis‐sensitive M2‐type TAMs levels. Combined with PD‐L1 blockade, it effectively prevented postoperative recurrence and metastasis of colorectal cancer.

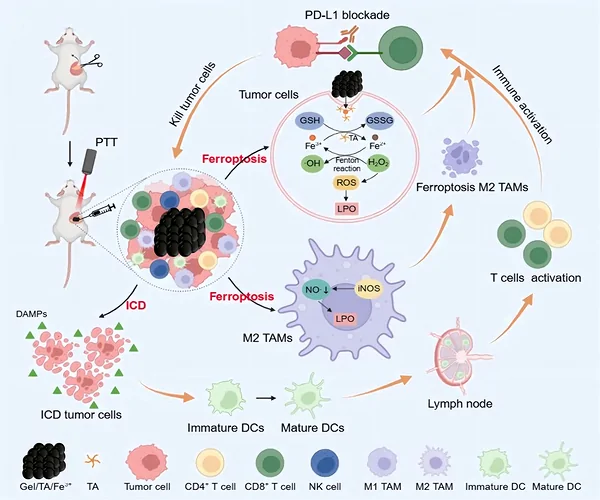

## 1 Introduction

Colorectal cancer (CRC) is a common malignant tumor of the digestive tract and a leading cause of cancer‐related mortality. Despite curative‐intent surgery, 30‐50% of CRC patients develop recurrence and metastasis, representing the main reason of death [1, 2]. In recent years, immunotherapy, particularly immune checkpoint blockade (ICB), has emerged as an effective postoperative treatment for recurrent or metastatic CRC due to the enhanced anti‐tumor immune responses by activating the host immune system [3, 4]. However, the poor immunogenicity of tumor cells and immunosuppressive tumor microenvironment (TME) results in low response rates to current immunotherapeutic approaches in patients with microsatellite‐stable and mismatch repair‐proficient (MSS/pMMR) CRC [5, 6, 7]. Consequently, promoting tumor immunogenicity and remodeling the TME are crucial in CRC postoperative immunotherapy, with the core to realize the immediate killing of residual tumor cells and long‐term maintenance of immune memory simultancously.

Inducing robust immunogenic cell death (ICD) is an effective strategy to elicit immune responses, which would promote the release of tumor‐associated antigens (TAAs) and damage‐associated molecular patterns (DAMPs), thereby driving dendritic cell (DC) maturation and T‐cell activation to evoke host immune responses [8, 9]. Numerous studies have shown that photothermal therapy (PTT) not only immediately kills tumor cells, but also induces ICD, thus inhibiting tumor growth, enhancing tumor immunogenicity and reversing immunosuppressive TME [10, 11, 12, 13]. Although PTT is a noninvasive, safe and controllable modality that could generate thermal energy under near‐infrared (NIR) irradiation to selectively ablate tumor cells locally [14], PTT‐induced ICD often results in a suboptimal immune response attributable to: (1) poor intratumoral accumulation and retention of photothermal agents; (2) difficulties in thermal dose control—insufficient heating fails to induce stable and efficient ICD, while overheating might rapidly degrade the released antigens and DAMPs, reducing antigen presentation efficiency [15]; and (3) the dynamic up‐regulation of immunosuppressive molecules within tumor tissues, such as PD‐L1, would suppress T‐cell activity, promote tumor immune evasion and encourage adaptive immune resistance [16, 17]. Apart from that, indiscriminate hyperthermia and intense irradiation inevitably induce severe damage to healthy tissues. Therefore, developing a precise and controllable PTT (mild PTT, ≤ 45°C) has achieved growing research attention, which could work directly on the postoperative residual tumor site [18].

To maximize the immunotherapeutic potential of mild PTT, it is essential to reverse immunosuppressive TME. Tumor‐associated macrophages (TAMs), especially the M2 subset, are pivotal in driving immunosuppressive TME and tumor progression [19]. Thus, decreasing the number of M2‐type TAMs or repolarizing them to the M1 phenotype could help reverse the immunosuppressive TME. Ferroptosis, a novel form of regulated cell death marked by intracellular iron buildup and lipid peroxidation (LPO), has shown significant immunomodulatory capacity in recent years [20, 21, 22, 23, 24]. Interestingly, compared with M1‐type TAMs, M2‐type TAMs are more vulnerable to ferroptosis, although the underlying mechanisms remain unclear. Therefore, combining mild PTT with ferroptosis might reshape the TME, producing robust and durable anti‐tumor immune effects [25]. It is worth noting that, conventional ferroptosis inducers, such as erastin and RSL3, suffer from poor aqueous solubility, short half‐life, and lack of tumor specificity [26]. Moreover, conventional administration methods are inadequate for achieving precise spatiotemporal coordination and synergism between ferroptosis and mild PTT, while advances in nanotechnology offer potential solutions, such as increased tumor accumulation mediated by the enhanced permeability and retention (EPR) effect and enable multimodal therapeutic synergy [27]. However, traditional intravenous nanoparticles delivery still faces challenges such as rapid immune clearance and the risk of systemic toxicity.

Gelatin, derived from the partial hydrolysis of collagen, offers excellent biocompatibility and biodegradability but is limited by poor mechanical properties. Tannic acid (TA) is a plant‐derived polyphenol exhibiting anticancer potential against multiple cancers [28]. As a natural phenolic crosslinking agent, TA could enhance the mechanical performance of gelatin. In addition, TA could also coordinate with different metal ions such as Fe^3+^, Cu^2+^ and Zn^2+^ to form metal‐polyphenol networks (MPNs). Especially, the chelates from TA and Fe^3+^ exhibit outstanding photothermal properties capable of triggering PTT. Furthermore, TA converts Fe^3+^ to Fe^2+^, initiating the Fenton reaction to induce ferroptosis. However, our previous research found that direct cross‐linking of gelatin with TA resulted in a “gum‐like” substance, which was unable to form injectable hydrogels.

To overcome these limitations, we designed an injectable, in situ‐adhesive tannic acid‐iron hydrogel (Gel/TA/Fe^3+^) with superior PTT and ferroptosis for providing a more efficient and safer strategy to enhance tumor inhibition and anti‐tumor immunity. First, gelatin microspheres were assembled with TA through hydrogen bonding to obtain injectability and adhesiveness. Then TA further chelated with Fe^3+^ to achieve the capacities of photothermal conversion and ferroptosis. Results showed that the gelatin microspheres and the TA system endow the hydrogel with injectability. Under NIR irradiation, Gel/TA/Fe^3+^ could maintain a temperature of 42‐45°C, effectively killing tumor cells and inducing ICD, which promoted DC maturation and T‐cell activation. Meanwhile, TA reduced Fe^3+^ to Fe^2+^, triggering Fenton reactions which generated substantial ROS and LPO accumulation, and induced ferroptosis in tumor cells and M2‐type TAMs, thereby reversing the immunosuppressive TME. When combined with αPD‐L1 therapy, it further suppressed postoperative recurrence and metastasis, transforming a weak immune response into a robust and enduring anti‐tumor immunity. This study might provide a facile and effective synergistic strategy for CRC postoperative treatment (Scheme 1).

**SCHEME 1:**
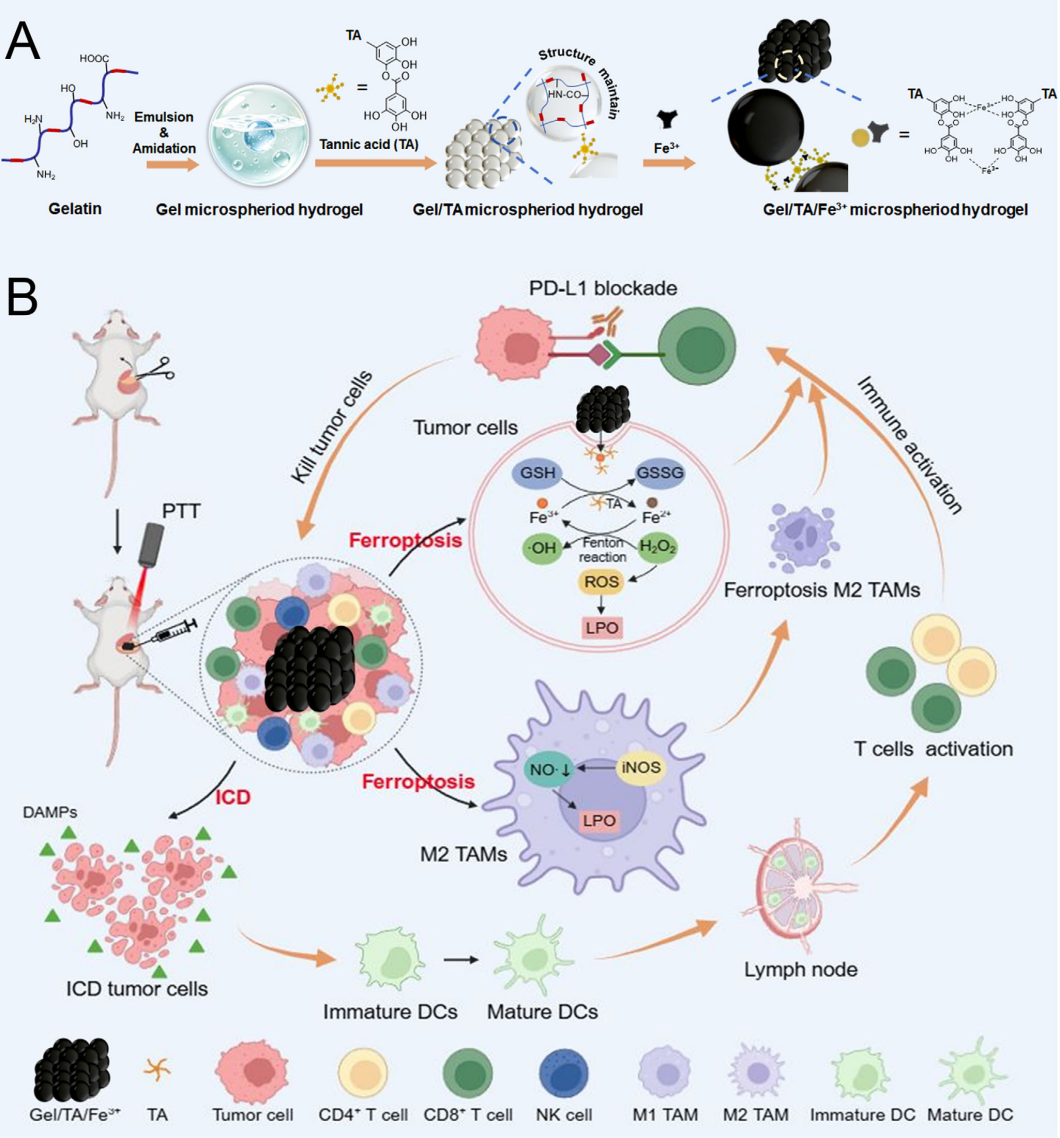
(A) The preparation procedure of Gel/TA/Fe^3+^. (B) Schematic illustration of Gel/TA/Fe^3+^ mechanism enhancing anti‐tumor immunity by inducing ICD via PTT and synergizing dual ferroptosis.

## 2 Materials and Methods

### 2.1 Materials

Gelatin was purchased from Sigma‐Aldrich (USA). EDC•HCl was obtained from GL Biochem Co., Ltd. (China). NHS and TA were purchased from Aladdin Reagent Co., Ltd. (China). Anhydrous Ferric Chloride (FeCl_3_) and Span 80 were obtained from Sinopharm Chemical Reagent Co., Ltd. (China). The CCK‐8 kit and Calcein‐AM/PI double‐staining kit were purchased from Dojindo (Japan). GPX4 antibody, Ki67 antibody, CD4 antibody, CD8 antibody, Granzyme B (GZMB) antibody, Alexa Fluor 488‐conjugated antibody, and CRT antibody were obtained from Abcam (UK). ACSL4 antibody was obtained from MCE (UK). The HMGB1 ELISA kit was purchased from Arigo Biolaboratories (Taiwan, China). ATP, GSSG, and ROS detection kits were obtained from Beyotime Biotechnology (China). The BODIPY^581/591^ C11 probe was purchased from Thermo Fisher Scientific (USA). CD11c‐PE, CD80‐APC, CD86‐FITC, CD3‐PE, CD4‐FITC, CD8‐APC, CD8‐Cy5.5, CD44‐FITC, CD62L‐APC, NKp46‐V450, CD11b‐PE, Gr‐1‐FITC, F4/80‐APC, CD86‐FITC, CD206‐FITC, CD86‐BV421, CD206‐PE‐Cy7, and iNOS‐APC were purchased from BioLegend (USA) and Invitrogen (USA). The TUNEL apoptosis detection kit was obtained from Roche (USA). The mouse anti‐PD‐L1 antibody and o‐Phenanthroline were purchased from Selleck Chemicals (USA). IL‐6, TNF‐α, IFN‐γ, IL‐12p70, and IL‐10 were obtained from Boyan Biotechnology Co., Ltd. (China). Alanine aminotransferase (ALT), aspartate aminotransferase (AST), creatinine (CRE), and blood urea nitrogen (BUN) assay kits were purchased from Nanjing Jiancheng Bioengineering Institute (China).

### 2.2 Cells and Animals

The mouse CRC cellline CT26, the human CRC cellline HCT116, and the mouse murine monocyte‐macrophage leukemia cell line RAW264.7 were obtained from the Cell Bank of the Chinese Academy of Sciences (China). CT26 and HCT116 cells were cultured in RPMI‐1640 medium, and RAW264.7 cells were cultured in DMEM, each supplemented with 10% fetal bovine serum and 1% penicillin‐streptomycin, at 37°C in a humidified incubator with 5% CO_2_.

Male Balb/c mice (6‐8 weeks old) were purchased from Shanghai JieSiJie Laboratory Animal Co., Ltd. (China). All animal experimental procedures were approved by the Ethics Committee of Putuo Hospital, Shanghai University of Traditional Chinese Medicine (approval no. DWEC‐A‐2025‐36‐1‐134).

### 2.3 Preparation of Gelatin Microspheres and Granular Hydrogels

Gelatin microspheres were prepared via an emulsion method. 0.2 g of gelatin was swollen in 2 mL of deionized water for 10 min, then heated at 50°C for 5 min to completely dissolve. 0.117 g of NHS was weighed and dissolved in the gelatin aqueous solution; separately, 0.228 g of EDC•HCl was weighed and dissolved in 1 mL of deionized water. 2 mL of Span 80 was poured into 30 mL of petroleum ether and emulsified at F‐level (24,000 rpm) for 30 s. The aforementioned gelatin aqueous solution and EDC•HCl solution were mixed uniformly and quickly poured into the emulsifying petroleum ether (water‐to‐oil ratio = 1:10), continuing emulsification for 2 min. The mixed emulsion was poured into a round‐bottom flask containing 120 mL of petroleum ether and stirred at 650 rpm for 9 h. The resulting white particles were washed several times with anhydrous ethanol. After vacuum drying for 24 h, white microgels were obtained.

Granular hydrogels (Gel/TA) were prepared by assembling gelatin microspheres with TA. 60 mg of gelatin microspheres were weighed and placed into a polytetrafluoroethylene (PTFE) mold. 500 µL of TA solutions at different concentrations were pipetted into the mold, followed by oscillation to ensure uniform mixing and a flat surface. The product was observed after different time intervals. Its mechanical properties were characterized, and its stability was tested to determine whether a granular gel had formed.

Subsequently, Gel/TA granular hydrogels were complexed with ferric ions to prepare granular hydrogels (Gel/TA/Fe^3+^). The assembled granular gels were immersed in FeCl_3_ solutions of different concentrations. After different immersion times, the products were taken out. The properties of the resulting products were observed. The products were placed into a syringe to observe whether they could be extruded and if they exhibited shear‐thinning behavior. Their photothermal conversion performance was characterized under infrared irradiation.

### 2.4 Rheological Tests

Gel storage modulus (G') and loss modulus (G'') measurements: Measurements were performed after the gel was assembled in a cylindrical mold. Frequency sweep experiments were conducted at room temperature from 0.01 to 100 Hz with 0.5% strain.

Gel breakpoint measurement: The values of G' and G'' were recorded, along with their crossover point, at room temperature with strain increasing from 0.01% to 500% and a fixed angular frequency (10 rad/s).

Gel shear‐thinning measurement: At room temperature, the shear rate was increased from 10^−3^ to 300 (1/s), observing the changes in sample viscosity and shear stress versus shear rate.

Gel self‐healing capability measurement: Alternating step strain scans were performed on hydrogel disks at a fixed angular frequency (10 rad/s). The initial G' and G'' of the hydrogel were recorded at 1% strain for 200 s. Then, a sudden strain of 200% was applied for 200 s to disrupt the hydrogel. Its self‐healing ability was observed.

### 2.5 Lap Shear Test

The prepared granular hydrogel was uniformly applied onto an area of 2.5 cm × 1.0 cm on the surfaces of two clean glass slides. The two slides were pressed together gently and left for 1 h. A lap shear test was then conducted using an electronic universal testing machine with a tensile rate of 50 mm/min. The force‐displacement curve was recorded, and the adhesive strength was calculated using the formula:

σ=F/A

where σ represents the adhesive strength in kPa, F represents the maximum force required to separate the glass slides in N, and A is the coated area of the adhesive (2.5 cm × 1.0 cm) in cm^2^.

### 2.6 Photothermal Test

The assembled granular gel was complexed in an FeCl_3_ solution for 15 min, removed, and excess water was absorbed with filter paper. It was then placed under a thermal imaging camera. Near‐infrared light with a wavelength of 808 nm was used for irradiation, and the temperature change over time was observed.

### 2.7 In vitro Cytotoxicity

CCK‐8 assay: CT26 or HCT116 cells were seeded into 96‐well plates (the lower chamber) at 1 × 10^5^/mL, and the Gel/TA/Fe^3+^ hydrogels were added to the upper chambers of the transwell system. The laser‐irradiated groups were exposed to 808 nm NIR (1 W/cm^2^) for 5 min. After 24 h, CCK‐8 reagent was added and incubated for 30 min, and absorbance was measured at 450 nm.

Calcein‐AM/PI double staining: CT26 cells were seeded into 24‐well plates (the lower chamber) at 2 × 10^5^/mL, and the Gel/TA/Fe^3+^ hydrogels were added to the upper chambers of the transwell system. The cells were exposed to NIR irradiation for 5 min. After 24 h, the cells were collected and stained with Calcein‐AM/PI working solution at 37°C for 15 min in the dark. The fluorescence images were observed using an inverted fluorescence microscope.

### 2.8 Detection of ICD

CRT immunofluorescence: CT26 cells were seeded into 24‐well plates (the lower chamber) at 2 × 10^5^/mL, and the Gel/TA/Fe^3+^ hydrogels were added to the upper chambers of the transwell system. The laser‐irradiated groups were exposed to 808 nm NIR (1 W/cm^2^) for 5 min. After 24 h, CT26 cells were digested and seeded into confocal dishes. The cells were fixed with 4% paraformaldehyde for 10 min, washed with PBS for three times, and permeabilized with 0.1% Triton X‐100 for 5 min. After blocking with 1% BSA for 1 h, cells were incubated with anti‐CRT antibody overnight. The following day, the cells were washed with PBS and stained with DAPI. Images were captured using a confocal laser scanning microscope (CLSM).

ATP and HMGB1 assays: CT26 cells were seeded into 24‐well plates (the lower chamber) at 2 × 10^5^/mL. The Gel/TA/Fe^3+^ hydrogels were added to the upper chambers of the transwell system and treated as before. After 24 h, cells were lysed and centrifuged, and the supernatants were collected for ATP detection using an ATP assay kit. For HMGB1 analysis, the supernatants were collected directly and analyzed using an HMGB1 ELISA kit.

### 2.9 In vitro Stimulation of DCs

Male Balb/c mice (4‐6 weeks old) were euthanized, and the femur and tibia were carefully removed. Bone marrow cells were obtained by flushing the marrow cavities with a syringe. Cells were cultured in RPMI‐1640 complete medium supplemented with 10 ng/mL GM‐CSF and 10 ng/mL IL‐4 to induce differentiation into immature DCs, which were seeded into 24‐well plates at 1 × 10^6^/mL. CT26 cells were seeded in the lower chamber, and the Gel/TA/Fe^3+^ hydrogels were added to the upper chambers of the transwell system and treated as before. After 24 h, the CT26 cells pre‐treated with Gel/TA/Fe^3+^ hydrogels were added to the upper chambers of the transwell system and co‐cultured with DCs in the lower chamber for 24 h. Afterward, the DCs were collected, incubated on ice with 2% Fc block for 5 min, and stained with CD11c‐PE, CD80‐APC, and CD86‐FITC for 30 min. Finally, DC maturation was analyzed by flow cytometry.

### 2.10 O‐Phenanthroline Color Reaction

All samples were incubated at room temperature for 2 h, then centrifuged at 12,000 rpm for 5 min. An equal volume of o‐phenanthroline colouring solution (0.2%, w/v) was added to the supernatant, mixed thoroughly, and allowed to stand at room temperature for 5 min. The color change was photographed against a white background (orange‐red indicates the presence of Fe^2+^).

### 2.11 Western Blotting

CT26 cells were seeded into 24‐well plates (the lower chamber) at 2 × 10^5^/mL. The Gel/TA/Fe^3+^ hydrogels were added to the upper chambers of the transwell system and treated as before. The cells were treated with ferroptosis inhibitor ferrostatin‐1 (Fer‐1, 10 µM). After 24 h, the cells were washed with PBS and lysed on ice for 30 min with RIPA buffer, and the lysates were centrifuged at 12,000 rpm for 15 min at 4°C to obtain total protein from the supernatant. Protein concentrations were determined using a BCA assay. Equal amounts of protein (20 µg) were separated on 15% SDS‐PAGE gels and transferred to PVDF membranes. The membranes were blocked with an 5% nonfat milk and incubated with anti‐GPX4 and anti‐ACSL4 antibody overnight at 4°C. After washing with TBST, the membranes were incubated with HRP‐conjugated secondary antibodies for 1 h and imaged using an ECL chemiluminescence.

### 2.12 Detection of GSSG, ROS, and LPO

CT26 cells in 24‐well plates were treated as described above. The cellular GSSG levels were measured using a GSSG assay kit. For analyzing ROS and LPO, cells in confocal dishes were washed with PBS, and incubated with DCFH‐DA (2.5 µM) or BODIPY^581/591^ C11 (1 µM) at 37°C for 30 min. After washed with PBS and stained with Hoechst 33342. Intracellular fluorescence was observed using a CLSM.

### 2.13 Induction of Macrophage Polarization

To compare the sensibility of macrophages to ferroptosis, RAW264.7 cells were first polarized into M1 and M2 macrophage phenotypes, respectively. RAW264.7 cells were seeded into 24‐well plates at 2 × 10^5^/mL. After adherence, the cells were incubated with 100 ng/mL lipopolysaccharide (LPS) for 48 h to induce M1 macrophages, or with 100 ng/mL IL‐4 in serum‐free medium for 48 h to induce M2 macrophages. The cells were collected, washed with PBS, and stained with CD11b‐PE, F4/80‐APC, CD86‐BV421, and CD206‐PE‐Cy7 for 30 min. The proportions of polarized macrophage subsets were analyzed by flow cytometry.

### 2.14 Flow Cytometry Analysis of iNOS

RAW264.7 cells were first polarized into M1 and M2 macrophage phenotypes, respectively. The cells were collected, washed with PBS. After surface staining with F4/80‐APC for 30 min, the cells were washed with stain buffer and centrifuged. The cell pellet was resuspended in freshly prepared fixation/permeabilization working solution and incubated for 60 min at 4 °C. After fixation, the cells were washed twice with permeabilization buffer, then stained with iNOS‐APC antibody for 45 min at 4 °C. Following two additional washes with permeabilization buffer, the cells were resuspended in stain buffer and analyzed by flow cytometry.

### 2.15 Subcutaneous Degradation of Gel/TA/Fe^3+^

The Gel/TA/Fe^3+^ was subcutaneously injected into the flank of Balb/c mice. One mouse was euthanized weekly to observe the skin area coated with Gel/TA/Fe^3+^ until completely resorbed. Skin tissues were collected for hematoxylin and eosin (H&E) staining.

### 2.16 In vivo Anti‐Tumor Efficacy

Subcutaneous recurrent CRC model: Luciferase‐labeled CT26 cells (200 µL, 1 × 10^7^/mL) were injected subcutaneously into the right flank of male Balb/c mice (4‐6 weeks old). When the tumor volume reached approximately 80 mm^3^, mice were randomized into five groups (n = 6): (1) saline control; (2) Gel/TA/Fe^3+^; (3) αPD‐L1; (4) Gel/TA/Fe^3+^ + laser (L); and (5) Gel/TA/Fe^3+^ + L + αPD‐L1. The mice were treated with tumor resection surgery, leaving a pea‐sized residual tumor to simulate postoperative remnants. For the hydrogel‐treated groups, Gel/TA/Fe^3+^ was applied to the surgical wound bed. For laser groups, the wound site was irradiated at 808 nm NIR irradiation (1 W/cm^2^) for 5 min on postoperative days 0, 2, and 4, maintaining temperature ≤ 45°C. For the αPD‐L1 groups, the mice received tail‐vein injections on postoperative days 1, 3, and 5. Starting on day 0, body weight and tumor size were measured every other day. Tumor volume (mm^3^) was calculated as 0.5 × length × width^2^. When tumor volume reached 1,500 mm^3^, mice were injected intraperitoneally with D‐luciferin potassium salt for in vivo bioluminescence imaging. Blood was collected from the retro‐orbital sinus, and the mice were euthanized; tumors, lymph nodes, and spleens were harvested, mechanically dissociated, and analyzed by flow cytometry to assess immune cell proportions. Major organs including hearts, livers, spleens, lungs, and kidneys were fixed in 4% paraformaldehyde for H&E staining.

Lung metastasis model: CT26 cells (200 µL, 1 × 10^7^/mL) were injected subcutaneously into the right flank of male Balb/c mice (4‐6 weeks old), followed by tail‐vein injection of CT26 cells (100 µL, 1 × 10^7^/mL). Mice were randomized into five groups (n = 6): (1) saline control; (2) Gel/TA/Fe^3+^; (3) αPD‐L1; (4) Gel/TA/Fe^3+^ + L; and (5) Gel/TA/Fe^3+^ + L + αPD‐L1. Procedures and treatments were as described above. On day 16, mice were euthanized, lungs were harvested, and the number and area of metastatic nodules were quantified.

### 2.17 Immunohistochemistry

Tumor tissues and heart, liver, spleen, lungs, and kidneys were collected, fixed in 4% paraformaldehyde, embedded in paraffin, and sectioned. Slides were stained with H&E and imaged under a light microscope.

Ki67 staining: After deparaffinization, the slides were immersed in 1× sodium citrate antigen retrieval buffer and subjected to microwave‐mediated antigen retrieval. The slides were treated with 3% H_2_O_2_ to block endogenous peroxidase and blocked with 5% BSA. Then, Ki67 antibody was added dropwise and incubated overnight at 4°C. After washed with PBS, biotin‐labeled anti‐rabbit IgG was added dropwise and incubated at 37°C for 30 min, followed by incubation with SABC for 30 min. Subsequently, the slides were stained with DAB chromogenic solutionand terminated by rinsing in water. Then, the slides were counterstained with hematoxylin for 10 s and photographed under a microscope.

### 2.18 Immunofluorescence and TUNEL Staining

Immunofluorescence: The slides were deparaffinized, subjected to antigen retrieval, and blocked as described above, then incubated with CD4 and CD8 primary antibodies overnight at 4°C. After washed with PBS, Alexa Fluor 488‐conjugated secondary antibody was applied and incubated for 1 h in the dark. The slides were washed with PBS, counterstained with DAPI for 10 min in the dark, and mounted with anti‐fade mounting medium. Fluorescence images were captured using a fluorescence microscope.

TUNEL staining: After deparaffinization, the slides were immersed in 1× sodium citrate antigen retrieval buffer and microwaved for antigen retrieval. The slides were fixed with a fixation solution and permeabilized with 0.1 M Tris‐HCl. The TUNEL reaction mixture was then applied and incubated at 37°C for 60 min in the dark. The slides were mounted with anti‐fade mounting medium and imaged using a fluorescence microscope.

### 2.19 Cell Flow Cytometry Analysis

Inguinal lymph nodes were minced and ground, then passed through a 70 µm cell strainer and centrifuged at 12,000 rpm for 10 min at 4°C. Cells were washed with PBS and adjusted to 1 × 10^7^/mL. The cells were incubated with 2% Fc block for 5 min. To analyze mature DCs in lymph nodes, cells were stained with CD11c‐PE, CD80‐APC, and CD86‐FITC for 30 min and analyzed by flow cytometry.

Spleens were minced and ground, filtered through a 70 µm strainer, and centrifuged at 1,200 rpm for 10 min at 4°C. Red blood cells were lysed with RBC lysis buffer on ice for 5 min. Cells were washed with PBS and adjusted to 1 × 10^7^/mL. The cells were incubated with 2% Fc block for 5 min. For T‐cell analysis, cells were stained with CD3‐PE, CD4‐FITC, and CD8‐APC for 30 min. For memory T‐cell analysis, cells were stained with CD3‐PE, CD8‐Cy5.5, CD44‐FITC, and CD62L‐APC for 30 min. For natural killer (NK) cell analysis, cells were stained with CD3‐PE and NKp46‐V450 for 30 min. For myeloid‐derived suppressor cell (MDSCs) analysis, cells were stained with CD11b‐PE and Gr‐1‐FITC for 30 min. Samples were analyzed by flow cytometry.

Tumors were minced and centrifuged at 1,200 rpm for 10 min at 4°C. Pellets were resuspended in 5 mL 1× collagenase IV (1 mg/mL) and 1 mL 1× DNase I (0.1 mg/mL), vortexed, and incubated on a horizontal shaker at 37°C and 140 rpm for 1 h. Following digestion, samples were centrifuged at 1,200 rpm for 5 min, resuspended in Hank's balanced salt solution (HBSS), and filtered through a 70 µm strainer. Cells were adjusted to 1 × 10^7^/mL. The cells were incubated with 2% Fc block for 5 min. For M1‐type TAMs analysis, cells were stained with CD11b‐PE, F4/80‐APC, and CD86‐FITC for 30 min; for M2‐type TAMs, cells were stained with CD11b‐PE, F4/80‐APC, and CD206‐FITC for 30 min. Samples were analyzed by flow cytometry.

### 2.20 Serum Cytokine Analysis

After trimming the whiskers of mice, blood was collected from the retro‐orbital venous plexus. Samples were allowed to clot for 30 min and centrifuged at 2,500 rpm for 20 min to obtain serum. IL‐6, TNF‐α, IFN‐γ, IL‐12p70, and IL‐10 levels in serum were quantified using ELISA kits.

### 2.21 Hepatic and Renal Function Assays

Serum and plasma samples were collected from each group. Levels of ALT, AST, CRE, and BUN were measured using assay kits.

### 2.22 Statistical Analysis

Statistical analyses were performed using GraphPad Prism 8. Data are presented as mean ± standard deviation (SD) from at least three independent experiments. Group differences were assessed by one‐way analysis of variance (ANOVA). Statistical significance in figures is indicated as follows: ^*^ *p* < 0.05, ^**^ *p* < 0.01, and ^***^ *p* < 0.001.

## 3 Results

### 3.1 Preparation and Evaluation of Gel/TA/Fe^3+^

N‐Hydroxysuccinimide (NHS) and 1‐Ethyl‐(3‐dimethylaminopropyl) carbodiimide Hydrochloride (EDC•HCl) have high activization efficiency and are widely used in amidation of gelatin hydrogels [29]. The carboxyl groups on aspartic acid and glutamic acid residues in the gelatin structure reacted with EDC to form an unstable O‐acylisourea intermediate, leading to cross‐linking of gelatin. To enhance the stability of the carbodiimide cross‐linking product, NHS was added to form a more stable amine‐reactive NHS ester. Observation under an upright microscope showed gelatin microspheres in the wet state. Scanning electron microscopy (SEM) observation of dried microspheres was shown in Figure 1A. The images revealed that the gelatin particulate gel has good sphericity. Sampling and measuring their diameters for particle size distribution statistics showed that the obtained gelatin microspheres had a relatively narrow size distribution, with an average diameter of about 10 µm (Figure 1B).

**FIGURE 1:**
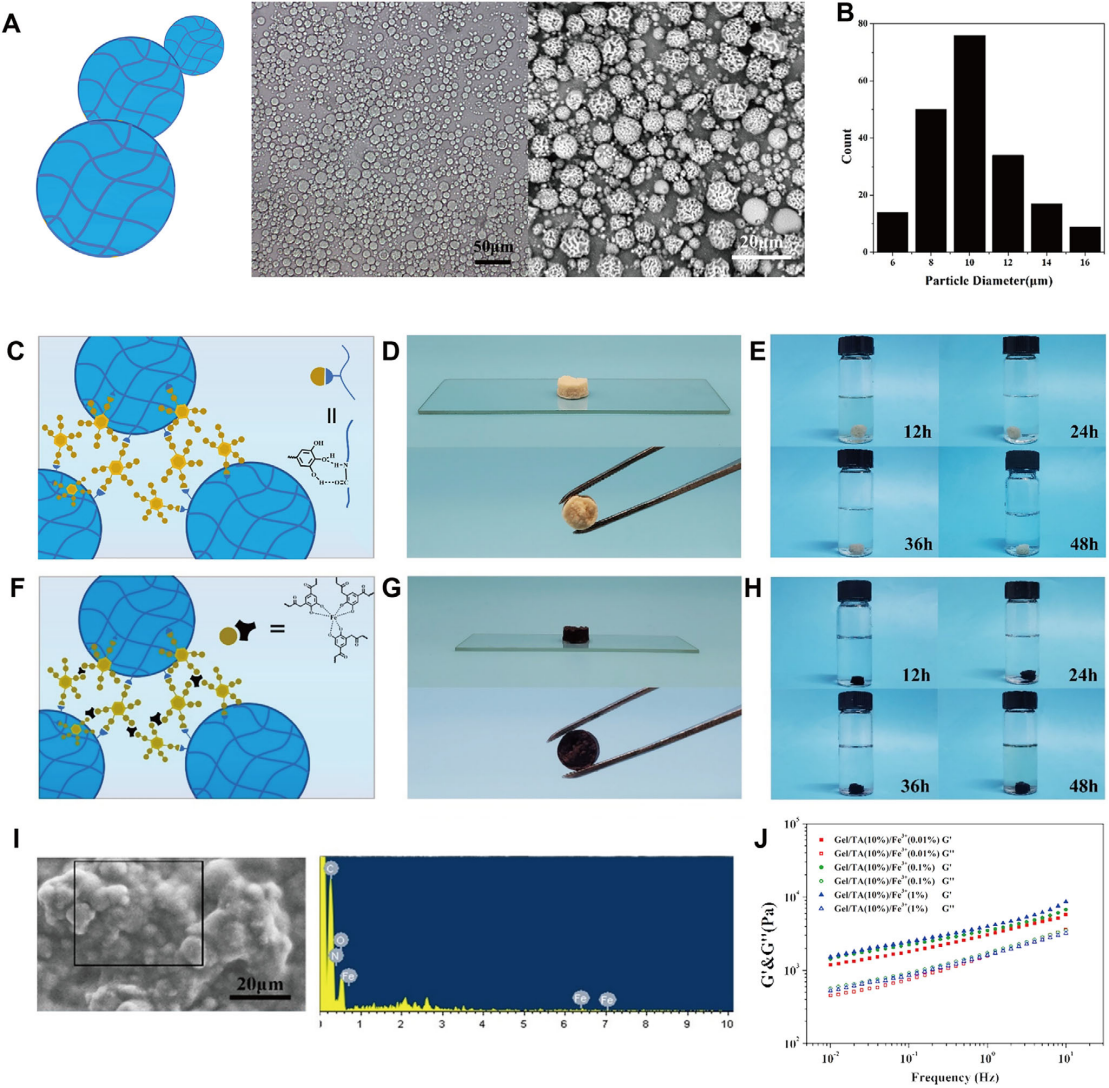
Preparation and evaluation of Gel/TA/Fe^3+^. (A) SEM image of gelatin microspheres. (B) Diameter statistics of gelatin microspheres. (C) Schematic diagram and (D) macrograph of Gel/TA system gel. (E) Stability of Gel/TA system gel in deionized water for 48 h. (F) Schematic diagram and (G) macrograph of Gel/TA/Fe^3+^ granular gel. (H) Stability of Gel/TA/Fe^3+^ granular gel in deionized water for 48 h. (I) EDS analysis of Gel/TA/Fe^3+^ granular gel. (J) Storage modulus (G') and loss modulus (G'') measurements from 0.01 to 100 Hz with 0.5% strain of Gel/TA/Fe^3+^.

TA contains abundant pyrogallol and catechol groups, which could form strong hydrogen bonds with various macromolecules. Dried gelatin microspheres were placed in a mold, and a TA solution was added. As shown in Figures 1C,D, hydrogen bonding could occur between TA and amino groups on the gelatin chains, resulting in a cross‐linked granular gel. The Gel/TA system could rapidly cure at room temperature. Due to hydrogen bonding, the Gel/TA system gel exhibits good stability. As shown in Figure 1E, the Gel/TA system gel was immersed in deionized water. After 48 h of immersion, no dispersion or collapse occurred. Interestingly, our previous research found that when dry gelatin powder was placed in a mold and TA solution was added, the gelatin cross‐linked with TA to form a “gum‐like” substance without spherical morphology (Figure S1A). After 48 h of immersion, no dispersion or collapse occurred (Figure S1B). Extrusion experiments showed that the gelatin and TA system could not be extruded through a needle, indicating a lack of injectability (Figure S1C). Gelatin is a water‐soluble protein with hydrophilic groups (amino and carboxyl groups) on its peptide chains, enabling easy dissolution in water. Upon cross‐linking with TA, the hydrophilic groups of gelatin would be shielded by the phenolic hydroxyl groups of TA through hydrogen bonding. This might result in a dense three‐dimensional network that restricted interactions between these hydrophilic groups and water molecules. Furthermore, the exposure of hydrophobic aromatic rings from TA on the surface of the network might further diminish the hydrophilicity of the material, ultimately leading to the formation of a water‐insoluble, gum‐like, elastic hydrophobic polymer. The challenges could be effectively addressed by assembling TA with gelatin microspheres into granular gels through hydrogen bonding interactions. The crosslinked network inherent to gelatin microspheres could restrict molecular chain movement. When interacting with TA, the gelatin microspheres could maintain an intact network structure, achieving injectability through microsphere bond slippage.

Furthermore, TA and Fe^3+^ could also form a stable supramolecular organometallic network [30]. As shown in Figure 1F,G, after the gelatin microspheres were assembled with TA, they were complexed in FeCl_3_ solutions of different concentrations. TA contains abundant phenolic hydroxyl groups, and Fe^3+^ could form stable chelates with the hydroxyl groups of TA to further stabilize the granular hydrogel. After treatment with the FeCl_3_ solution, the sample color changed from milky white to black. Similarly, the Gel/TA/Fe^3+^ system gel still exhibited good stability. Immersing the Gel/TA/Fe^3+^ granular gel in deionized water, PBS with 10% FBS, and collagenase IV for 48 h resulted in no dispersion or collapse (Figure 1H and Figure S2). EDS analysis of the Gel/TA/Fe^3+^ granular gel (Figure 1I) confirmed the presence of iron elements in the gel. Rheological test results showed that the storage modulus G' of the granular hydrogels with different compositions was greater than their loss modulus G'', confirming that the granular gels possess the elasticity of a bulk hydrogel (Figure 1J). Different concentrations of Fe^3+^ showed an influence on the cross‐linking performance, which further affected the mechanical property levels. Compared to the Gel/TA(10%)/Fe^3+^(0.01%) granular gel, the G' value of the Gel/TA(10%)/Fe^3+^(1%) granular gel increased from 5401 Pa to 8653 Pa at 0.1 Hz. The G' value of the Gel/TA(10%)/Fe^3+^(0.1%) granular gel was not significantly different from that of the Gel/TA(10%)/Fe^3+^(1%) granular gel.

### 3.2 Characterization of Gel/TA/Fe^3+^

Although the granular gels exhibit properties of bulk hydrogels, due to the reversible supramolecular interactions within the granular gel, a “solid‐liquid” transition could occur under certain shear forces, resulting in flowability (Figure 2A). Meanwhile, when the TA concentration was maintained at 10%, compared to the Gel/TA(10%)/Fe^3+^(0.01%) gel which fractured at a strain amplitude of 2.04%, the values for Gel/TA(10%)/Fe^3+^(0.1%) and Gel/TA(10%)/Fe^3+^(1%) were 4.04% and 5.07% respectively, showing a slight increase in the breakpoint strain amplitude, which could be attributed to the presence of metal coordination. TA and Fe^3+^ improved the mechanical properties of the gels. As shown in Figure 2B, the viscosity of granular gels with different Fe^3+^ concentrations decreased as the shear rate increased, indicating that all the obtained Gel/TA/Fe^3+^ gel systems possess shear‐thinning characteristics, which aided injection operations. According to Figure 2C, after undergoing multiple “low strain‐high strain” cycles, the G' of the granular hydrogel did not decrease significantly, indicating that the granular hydrogel has good self‐healing capability.

**FIGURE 2:**
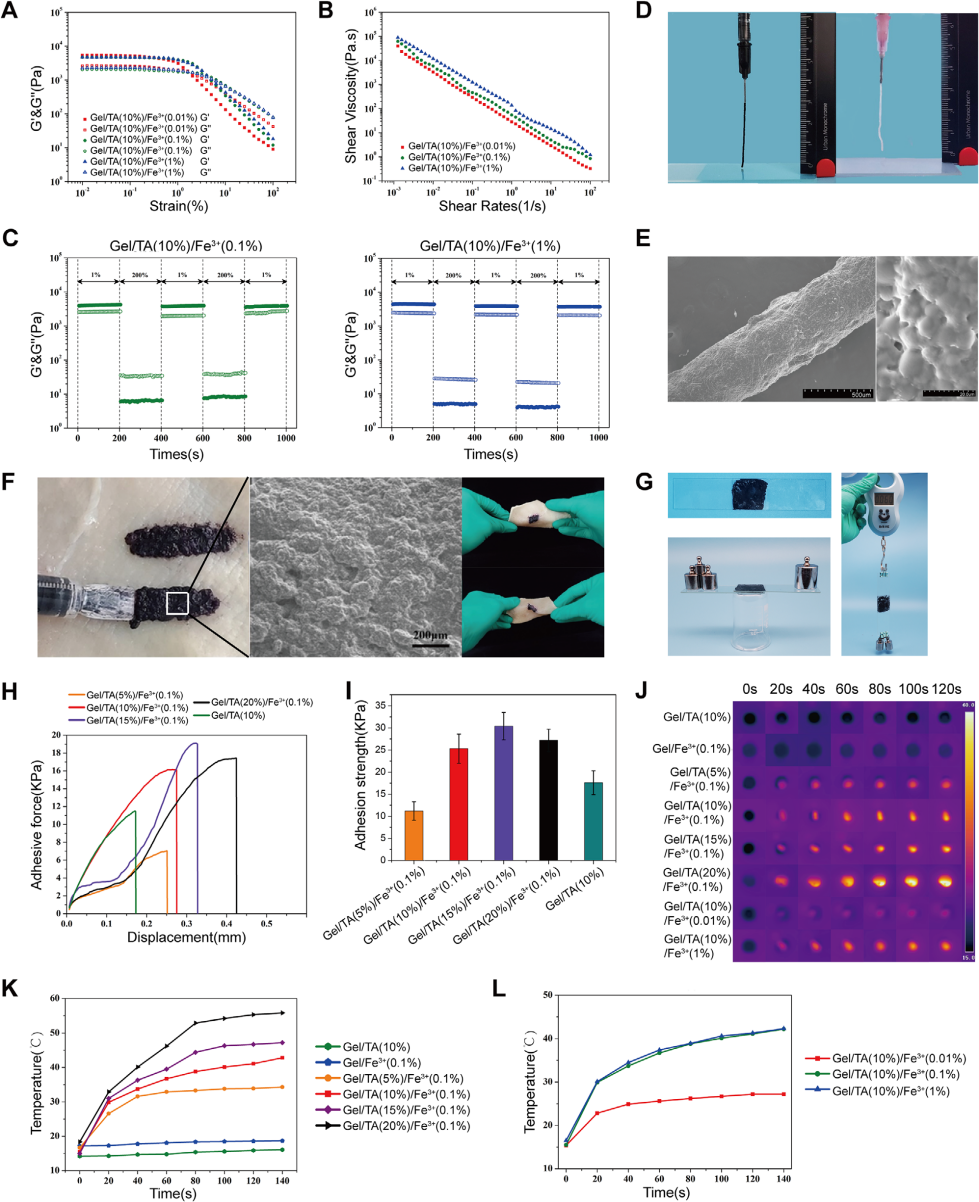
Characterization of Gel/TA/Fe^3+^. (A) Storage modulus (G') and loss modulus (G'') with strain increasing from 0.01% to 500% and a fixed angular frequency (10 rad/s) of Gel/TA/Fe^3+^. (B) Shear viscosity with increasing shear rates from 10^−3^ to 300 (1/s) of Gel/TA/Fe^3+^. (C) Evaluation of self‐healing capability of Gel/TA/Fe^3+^ under strains of 1% and 200%. (D) Observation of the extrusion properties of Gel/TA/Fe^3+^ (left) and Gel/TA (right). (E) SEM image of the extruded filaments. (F) Observation of Gel/TA/Fe^3+^ adhering to pig skin. (G) Macroscopic adhesion tests and lap shear tests of Gel/TA/Fe^3+^. (H, I) Displacement‐adhesive strength of Gel/TA/Fe^3+^ and Gel/TA. (J) Photothermal images and (K, L) heating curves of Gel/TA/Fe^3+^ and Gel/TA at different time points under NIR irradiation.

Extrusion experiments demonstrated that both Gel/TA/Fe^3+^ granular gels and Gel/TA granular gels without Fe^3+^ are injectable. They could be continuously extruded through a 20 G needle, maintaining the extruded strand without breakage (Figure 2D). SEM images of the extruded filaments showed that due to the presence of TA and the chelation of Fe^3+^, the contours of the gelatin particles became blurred, but it was still possible to see that the extruded filaments were composed of a large number of densely packed particles (Figure 2E).

However, it was worth noting that the component content significantly affects the formation and extrudability of the granular gels, as shown in Table 1 and Table 2. On one hand, when the TA concentration was too low (≤4%), it could not assemble the gelatin microspheres into a stable system, and the structure collapsed quickly after removal from the mold. Higher TA concentrations made gel formation easier and the gel structure more stable. However, the high stability resulting from excessively high TA concentrations had drawbacks. Compared to TA concentrations of 5% and 10%, gelatin microspheres assembled with 15% and 20% TA were more difficult to extrude. On the other hand, the concentration of the FeCl_3_ solution had no significant influence on the extrudability of the already formed gel system.

**TABLE 1:** Effect of TA Concentration and Assembly Time on Gel Formation.

Sample No.	Gelatin Microspheres (mg)	TA Concentration (%)	Reaction Time (h)	Cured	Extrudable
1	60	2	0.5	×	×
2	60	4	0.5	×	×
3	60	5	0.5	×	×
4	60	10	0.5	×	×
5	60	15	0.5	×	×
6	60	20	0.5	√	×
7	60	2	1	×	×
8	60	4	1	×	×
9	60	5	1	√	√
10	60	10	1	√	√
11	60	15	1	√	√
12	60	20	1	√	√
13	60	2	1.5	×	×
14	60	4	1.5	×	×
15	60	5	1.5	√	√
16	60	10	1.5	√	√
17	60	15	1.5	√	√
18	60	20	1.5	√	√

**TABLE 2:** Effect of FeCl_3_ Concentration on Gel Formation.

Sample No.	TA Concentration (%)	FeCl_3_ Solution Concentration (%)	Reaction Time (min)	Extrudable
1	5	0.01	5	√
2	5	0.1	5	√
3	5	1	5	√
4	10	0.01	5	√
5	10	0.1	5	√
6	10	1	5	√
7	5	0.01	10	√
8	5	0.1	10	√
9	5	1	10	√
10	10	0.01	10	√
11	10	0.1	10	√
12	10	1	10	√
13	5	0.01	20	√
14	5	0.1	20	√
15	5	1	20	√
16	10	0.01	20	√
17	10	0.1	20	√
18	10	1	20	√

To demonstrate the coating performance of the gel, wet pig skin was used to mimic human skin, and the gel was extruded and coated onto it in a strip (Figure 2F). The gel adhered well to the skin. Bending and twisting the coated area did not cause detachment, proving that it can firmly adhere to the pig skin.

To further evaluate the tissue adhesion performance of the gel, macroscopic adhesion tests and lap shear tests were conducted. As shown in Figure 2G, a 5% gelatin solution was uniformly applied to the surfaces of two glass slides and dried. The Gel/TA/Fe^3+^ granular gel was extruded and applied onto one slide, and the two slides were pressed together. When forces were applied laterally and longitudinally at the adhesion site, the two glass plates remained well‐bonded by the gel. A simple weight hanging test was used to preliminarily evaluate the adhesion performance of the Gel/TA/Fe^3+^ granular gel. The results showed that the adhesive strength could support a 50 g weight on the side of the glass plates and a 60 g weight on the bottom side of the glass plate, indicating good adhesion properties of the granular gel. The adhesive strength was evaluated by lap shear tensile tests. Figure 2H,I showed the adhesive strength of the tested samples. Granular gels without Fe^3+^ also exhibited adhesion. The adhesive strength of Gel/TA(10%) was approximately ∼17 kPa. When iron ions were not added to the gel system, the adhesive strength of the granular hydrogel increased, possibly due to improved cohesion. The adhesive strength of Gel/TA(10%)/Fe^3+^(0.1%) was approximately ∼25 kPa, significantly higher than that of Gel/TA(10%). Simultaneously, as the TA content increased, the adhesive strength of the granular hydrogel also increased. The adhesive strengths of Gel/TA(5%)/Fe^3+^(0.1%), Gel/TA(10%)/Fe^3+^(0.1%), and Gel/TA(15%)/Fe^3+^(0.1%) were approximately ∼11, ∼25, and ∼30 kPa, respectively. As the TA concentration in the gel continued to increase, the adhesive strength of the Gel/TA(20%)/Fe^3+^(0.1%) granular gel slightly decreased to approximately ∼27 kPa.

TA/Fe^3+^, acting as a photothermal agent, has a good photothermal conversion effect. As shown in Figure 2J–L, the photothermal capacity of the Gel/TA/Fe^3+^ system gel was measured by irradiating each sample with an NIR laser (808 nm) for 2 min. For the Gel/TA and Gel/Fe^3+^ systems, no significant temperature increase was observed after irradiation. However, in the Gel/TA/Fe^3+^ system, the photothermal response amplitude of the gel was greatly enhanced. By increasing the concentration of TA or Fe^3+^, the maximum achievable temperature was significantly increased. After 2 min of NIR irradiation, the temperature of the Gel/TA(5%)/Fe^3+^(0.1%) gel increased by about 20°C. Gel/TA(10%)/Fe^3+^(0.1%) and Gel/TA(15%)/Fe^3+^(0.1%) showed temperature increases of about 30°C. Furthermore, granular gels with different Fe^3+^ concentrations also displayed different photothermal conversion capabilities. Compared to Gel/TA(10%)/Fe^3+^(0.01%), the maximum temperature achievable by Gel/TA(10%)/Fe^3+^(0.1%) increased by 15°C. However, further increasing the Fe^3+^ concentration had little effect on the photothermal conversion capability; the temperature rise curve of Gel/TA(10%)/Fe^3+^(1%) basically overlapped with that of Gel/TA(10%)/Fe^3+^(0.1%). All the above results indicated that the Gel/TA/Fe^3+^ system gel had excellent photothermal conversion capability, with the maximum temperature not exceeding 60°C. After 5 min of NIR irradiation, the temperature of the Gel/TA(10%)/Fe^3+^(0.1%) gel gradually rose to 45°C (Figure S3). For the subsequent experiments, this ratio was ultimately selected.

### 3.3 Gel/TA/Fe^3+^ Induces ICD in Colorectal Cancer Cells and DC Maturation In vitro

As shown in Figure 3A, the in vitro interaction between hydrogels and colorectal cancer cells was investigated using a Transwell system. Colorectal cancer cells were seeded in the lower chamber, while the Gel/TA/Fe^3+^ was placed in the upper chamber. To assess the in vitro anti‐tumor activity, we used CCK‐8 assays to examine the cytotoxic effects of different concentrations of Gel/TA/Fe^3+^ combined with PTT on CT26 and HCT116 cells. Cells incubated with the Gel/TA/Fe^3+^ without NIR irradiation showed almost no obvious toxicity, which proved that Gel/TA/Fe^3+^ have good biocompatibility. However, the cytotoxicity of Gel/TA/Fe^3+^ demonstrated a concentration‐dependent manner upon 808 nm laser irradiation (Figure 3B,C). The formulation Gel/TA(10%)/Fe^3+^(0.1%) maintained a stable temperature around 45°C, which provides adequate photothermal effect while avoiding thermal damage to cells (Figure S4). Hence, this ratio was adopted for subsequent cell experiments. Subsequently, the cytotoxic effects of different concentrations of Gel/TA/Fe^3+^ on CT26 cells, with or without laser irradiation, were assessed using Calcein AM/PI double staining (Figure 3D,E and Figure S5). Consistent with the CCK‐8 assay results, a remarkable increase in PI‐positive (dead) cells was observed upon laser irradiation, demonstrating that Gel/TA/Fe^3+^‐mediated mild PTT exhibits anti‐tumor activity effectively.

**FIGURE 3:**
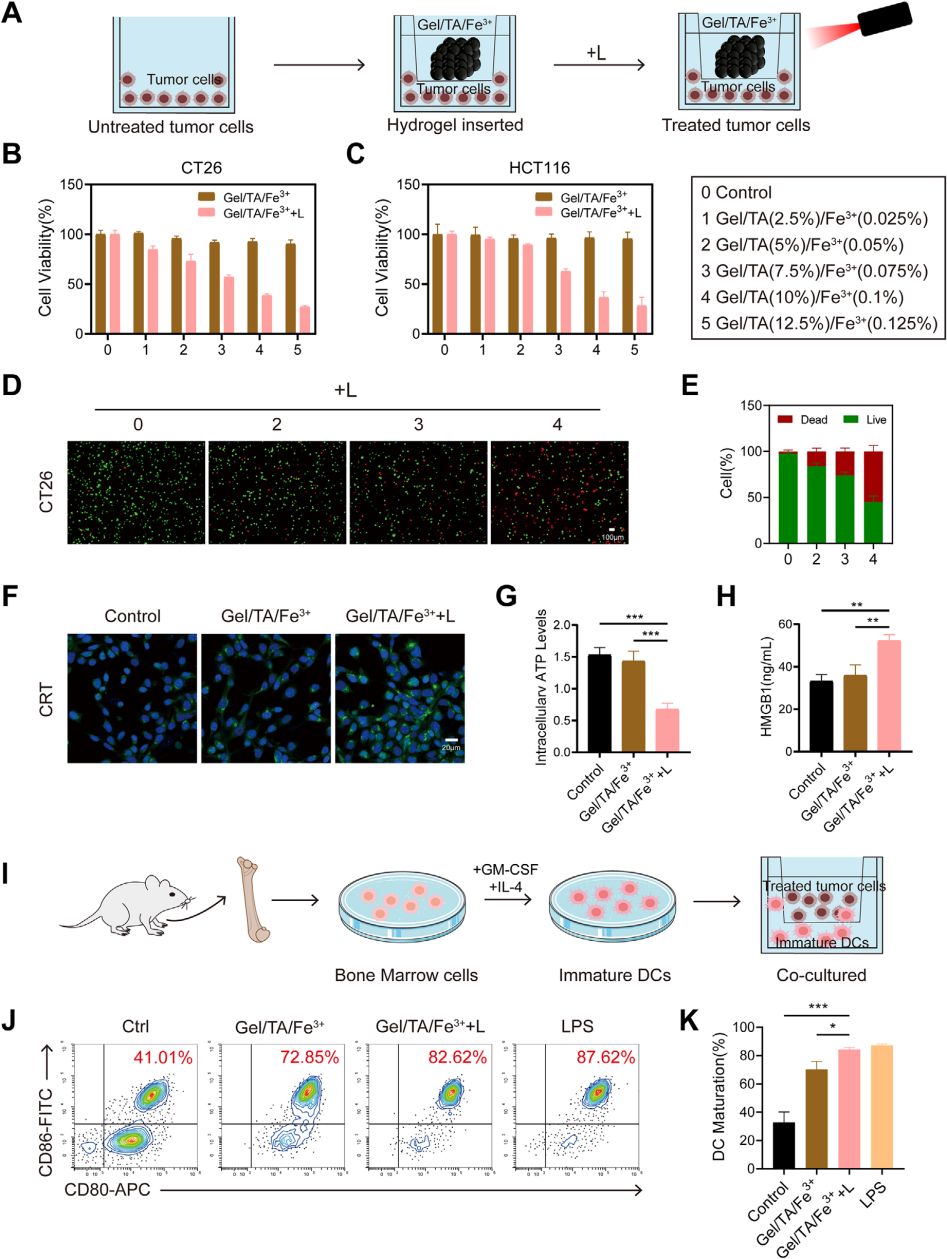
Gel/TA/Fe^3+^ induces ICD in colorectal cancer cells and DC maturation in vitro. (A) Schematic illustration of the in vitro system for Gel/TA/Fe^3^^+^ interacting with colorectal cancer cells. (B, C) Viability of CT26 and HCT116 cells after treatment with different concentrations of Gel/TA/Fe^3+^ without or with NIR irradiation for 24 h. (D, E) Fluorescence images of Calcein‐AM/PI double staining of CT26 cells after treatment with Gel/TA/Fe^3+^ with NIR irradiation. Scale bar = 100 µm. (F) CLSM images of CRT exposure in CT26 cells after treatment with Gel/TA/Fe^3+^ and NIR irradiation (CRT, green fluorescence; DAPI, blue fluorescence). Scale bar = 20 µm. (G) Extracellular HMGB1 and (H) intracellular ATP levels of CT26 cells after the indicated treatments (mean ± SD; n = 3). (I) Schematic diagram of the co‐culture system used to induce DC maturation. (J) Flow cytometry analysis of DC maturation following various treatments. (K) Quantitative analysis of DC maturation (mean ± SD; n = 3). *p < 0.05, **p < 0.01 and ***p < 0.001.

ICD is characterized by the release of DAMPs, including signaling molecules such as calreticulin (CRT), adenosine triphosphate (ATP), and high mobility group box 1 (HMGB1). These DAMPs are recognized by pattern recognition receptors (PRRs) on the surface of DCs, promoting DC maturation and T‐cell activation, which initiates immune responses [31]. We further explored whether Gel/TA/Fe^3+^ could induce ICD in colorectal cancer cells. As shown in Figure 3F, the Gel/TA/Fe^3+^ + L group presented the most significant CRT exposure (green fluorescence) on the surface of CT26 cells. In parallel, the Gel/TA/Fe^3+^ + L group exhibited the lowest intracellular ATP levels and the highest extracellular HMGB1 levels (Figure 3G,H). These results demonstrated that Gel/TA/Fe^3+^‐mediated mild PTT can induce ICD in CRC cells.

DCs are the most potent antigen‐presenting cells in vivo. Upon stimulation by DAMPs, mature DCs efficiently present tumor‐specific antigens to naive T cells, thereby activating CD8^+^ T cells and initiating anti‐tumor immune responses [32, 33]. To verify whether Gel/TA/Fe^3+^‐induced ICD promotes DC maturation and subsequent immune activation, marrow‐derived DCs were co‐cultured with CT26 cells subjected to different pretreatments (Figure 3I). After 24 h, DC maturation was analyzed by flow cytometry. As shown in Figure 3J,K, Gel/TA/Fe^3+^ treatment increased the proportion of mature DCs (70.30 ± 5.49%) relative to the control group (32.68 ± 7.49%), likely due to the induction of ferroptosis. The Gel/TA/Fe^3+^+L group further increased mature DCs to 84.25 ± 1.43%, approaching the level observed with LPS stimulation (87.21 ± 0.95%). These results indicated that Gel/TA/Fe^3+^ mediated mild PTT could effectively induce DC maturation in vitro and activate anti‐tumor immunity.

### 3.4 Gel/TA/Fe^3+^ Induces Colorectal Cancer Cells and M2 Macrophage Ferroptosis In vitro

Ferroptosis is an iron‐dependent form of cell death driven by the excessive accumulation of LPO via Fenton chemistry, leading to membrane rupture and cell death. This process is characterized by increased ROS, depletion of glutathione (GSH), and reduced activity of glutathione peroxidase 4 (GPX4) [26].

After the dissociation of Gel/TA/Fe^3+^ in the acidic TME, TA could reduce Fe^3+^ to Fe^2+^, leading to excessive accumulation of intracellular iron ions. To quantify this process, we measured the Fe release kinetics using ICP‐MS, which showed that acidic microenvironment could mildly accelerate the Fe release compared with the neutral environment due to the dissociation of TA‐Fe^3+^ chelation (Figure S6). Meanwhile, the Fe^3+^/Fe^2+^ conversion was assessed by o‐Phenanthroline color reaction. The results showed more Fe^3+^ and TA were released to participate Fenton reaction due to the TA‐Fe^3+^ dissociation via acidic environment and photothermal conversion. Thus in pH5.5 + Gel/TA/Fe^3+^ + L group, a deeper orange‐red was observed compared with pH7.4 + Gel/TA/Fe^3+^ and pH5.5 + Gel/TA/Fe^3+^ groups (Figure S7). To verify whether Gel/TA/Fe^3+^ can induce ferroptosis in colorectal cancer cells in vitro, the expression of ROS and LPO in CT26 cells was detected using DCFH‐DA and BODIPY^581/591^ C11 probes respectively. As shown in Figure 4A, green fluorescent signals were observed in the Gel/TA/Fe^3+^ and Gel/TA/Fe^3+^ + L groups, with the strongest signal in the Gel/TA/Fe^3+^ + L group. However, compared with the Gel/TA/Fe^3+^ + L group, the green fluorescence intensity in the Gel/TA/Fe^3+^ + L + Fer‐1 (ferroptosis inhibitor) group significantly decreased. This indicated that Gel/TA/Fe^3+^ promotes the production of ROS and LPO, and mild PTT further accelerates ferroptosis. Fe^2+^ generates phospholipid hydroperoxides (PLOOH) through the Fenton reaction, while GPX4 protects cells from ferroptosis by reducing PLOOH to the corresponding alcohol (PLOH) [24, 34]. In addition, the expression level of GPX4 and ACSL4 in CT26 cells was detected by Western blot assay. As shown in Figure 4B, the GPX4 level was notably reduced in the Gel/TA/Fe^3+^ and Gel/TA/Fe^3+^ + L groups and increased in the Gel/TA/Fe^3+^ + L + Fer‐1 group. Conversely, ACSL4 expression was significantly upregulated in the Gel/TA/Fe^3+^ and Gel/TA/Fe^3+^ + L groups, and this upregulation was reversed by Fer‐1 (Figure S8). These results suggested that Gel/TA/Fe^3+^ may inhibit GPX4 while activating ACSL4, leading to the accumulation of LPO. GSH, the substrate for GPX4, can be oxidized by ROS to form oxidized glutathione (GSSG). The GSSG detection kit was used to measure GSSG levels. As shown in Figure 4C, the intracellular concentration of GSSG in the Gel/TA/Fe^3+^ and Gel/TA/Fe^3+^ + L groups increased significantly due to GSH consumption. These results indicated that Gel/TA/Fe^3+^ under NIR irradiation effectively consume GSH and generates ROS, thus inducing ferroptosis in CRC cells.

**FIGURE 4:**
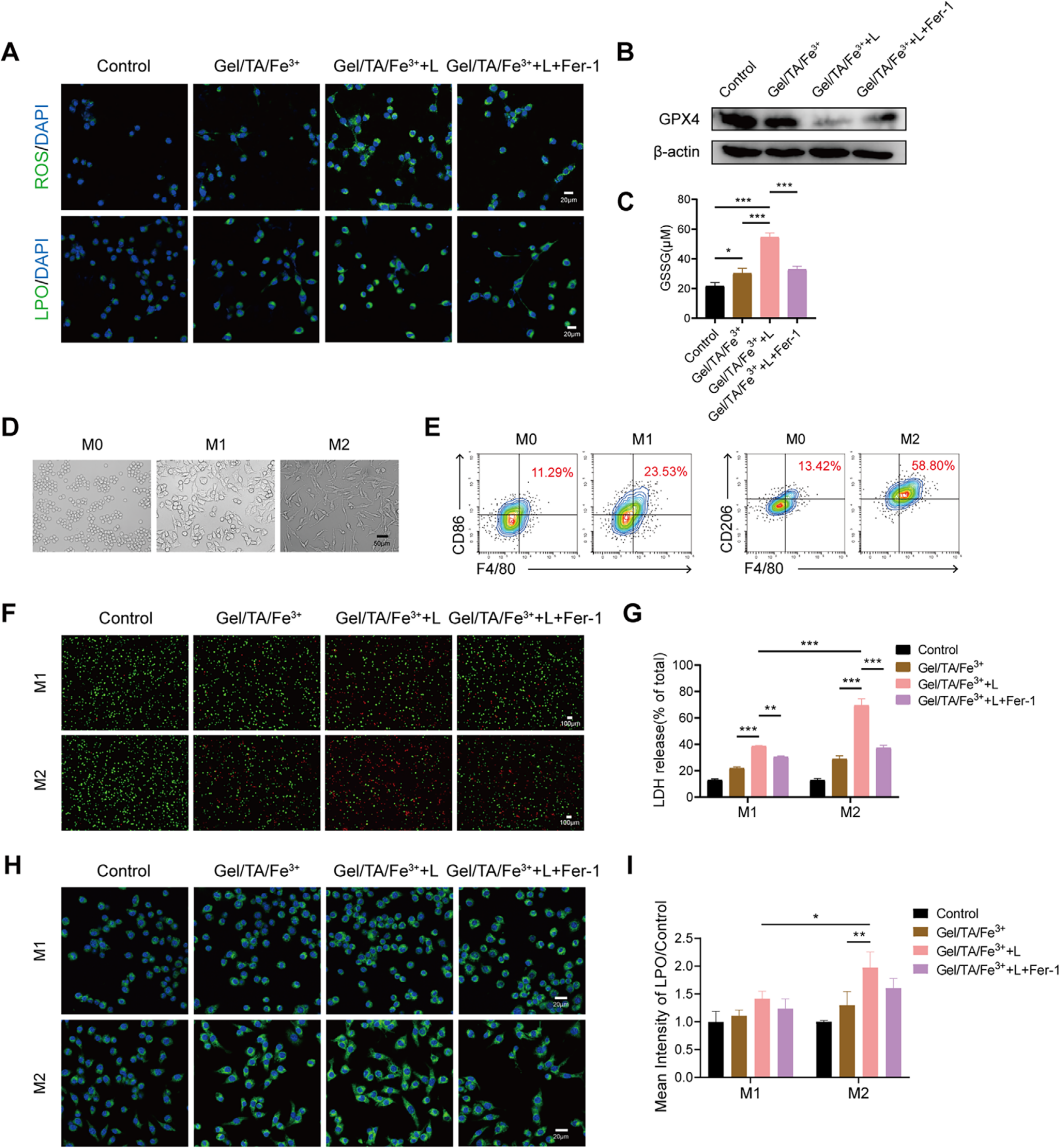
Gel/TA/Fe^3+^ induces ferroptosis in colorectal cancer cells and M2 macrophages in vitro. (A) Fluorescence images of ROS and LPO in CT26 cells after treatment (ROS and LPO, green fluorescence; Hoechst, blue fluorescence). Scale bar = 20 µm. (B) Western blot analysis of GPX4 protein expression in CT26 cells after treatment. (C) Intracellular concentration of GSSG in CT26 cells after treatment (mean ± SD; n = 3). (D) Representative microscopy images of M0, M1, and M2 macrophages. Scale bar = 50 µm. (E) Flow cytometry analysis of F4/80^+^ CD86^+^ and F4/80^+^ CD206^+^ macrophages. (F) Fluorescence images of Calcein‐AM/PI double staining of M1 and M2 macrophages after treatment. (G) LDH release assay of M1 and M2 macrophages after treatments (mean ± SD; n = 3). (H) Fluorescence images of LPO in M1 and M2 macrophages after treatment (LPO, green fluorescence; Hoechst, blue fluorescence). Scale bar = 20 µm. (H) Mean fluorescence intensity of LPO (mean ± SD; n = 3). *p < 0.05, **p < 0.01 and ***p < 0.001.

We further investigated the role of Gel/TA/Fe^3+^ in regulating tumor‐associated macrophages (TAMs). Initially, as shown in Figure 4D, the morphological characteristics of M1 and M2 macrophages confirmed their successful induction. Flow cytometry analysis revealed that compared with M0 macrophages, the proportion of F4/80^+^CD86^+^ cells was significantly increased in M1 macrophages, while the proportion of F4/80^+^CD206^+^ cells was markedly elevated in M2 macrophages, indicating the successful polarization of M1 and M2 macrophages, respectively (Figure 4E). Calcein‐AM (green fluorescence) and PI (red fluorescence) were used to label M1 and M2 macrophages, allowing visualization of viable and dead cell distributions. Results from the live/dead staining assay further demonstrated that nearly all M2 macrophages succumbed to Gel/TA/Fe^3+^‐induced ferroptosis, whereas the majority of M1 macrophages remained viable (Figure 4F). To further evaluate the capacity of Gel/TA/Fe^3+^ under laser to induce ferroptosis on TAMs, a standard lactate dehydrogenase (LDH) release assay was performed. As illustrated in Figure 4G, the LDH release rate of M2 macrophages reached 69.51 ± 4.97% under NIR irradiation, which was significantly higher than that of M1 macrophages (38.70 ± 0.47%). These results demonstrated that M2 macrophages were more sensitive to Gel/TA/Fe^3+^‐induced ferroptosis, and this effect was more pronounced following NIR irradiation. The expression levels of inducible nitric oxide synthase (iNOS) in different types of macrophages were evaluated using flow cytometry. M1 macrophages show much higher expression of iNOS than M0 and M2 macrophage (Figure S9). iNOS is the key enzyme involved in the catalytic production of nitric oxide (NO•) [35]. NO• can scavenge lipid radicals (L•, LOO•) to form nitrated lipid products (LNO_2_), thereby slowing down the lipid peroxidation chain reaction [36]. Then, we further evaluated the accumulation of LPO induced by Gel/TA/Fe^3+^ + L in M1 and M2 macrophages, a key event in ferroptosis induction. As anticipated, intense green fluorescence from Lipfluo was observed in M2 macrophages via CLSM, indicating substantial Gel/TA/Fe^3+^ + L‐induced LPO accumulation (Figure 4H,I). These findings collectively suggest that M2 macrophages are more susceptible to Gel/TA/Fe^3+^ + L‐induced ferroptosis, while M1 macrophages display relative resistance. In conclusion, Gel/TA/Fe^3+^‐mediated mild PTT can selectively eliminate M2 macrophages by inducing ferroptosis in macrophages, for further attenuating immunosuppressive TME in vivo, thus exerting a potent anti‐tumor immune effect.

### 3.5 Biodegradation of Gel/TA/Fe^3+^ In Vivo

We further developed an injectable Gel/TA/Fe^3+^ hydrogel that can be applied to post‐surgical wound sites. The biodegradability and biocompatibility of hydrogels are critical for clinical applications. To evaluate the in vivo degradation of Gel/TA/Fe^3+^, the hydrogel was placed through extrusion subcutaneously injected into the flank of mice. As shown in Figure 5A, Gel/TA/Fe^3+^ degraded gradually and was completely resorbed at 6 weeks. Hematoxylin and eosin (H&E) staining revealed no skin damage, demonstrating that Gel/TA/Fe^3+^ exhibits excellent in vivo biodegradability and biocompatibility (Figure 5B).

**FIGURE 5:**
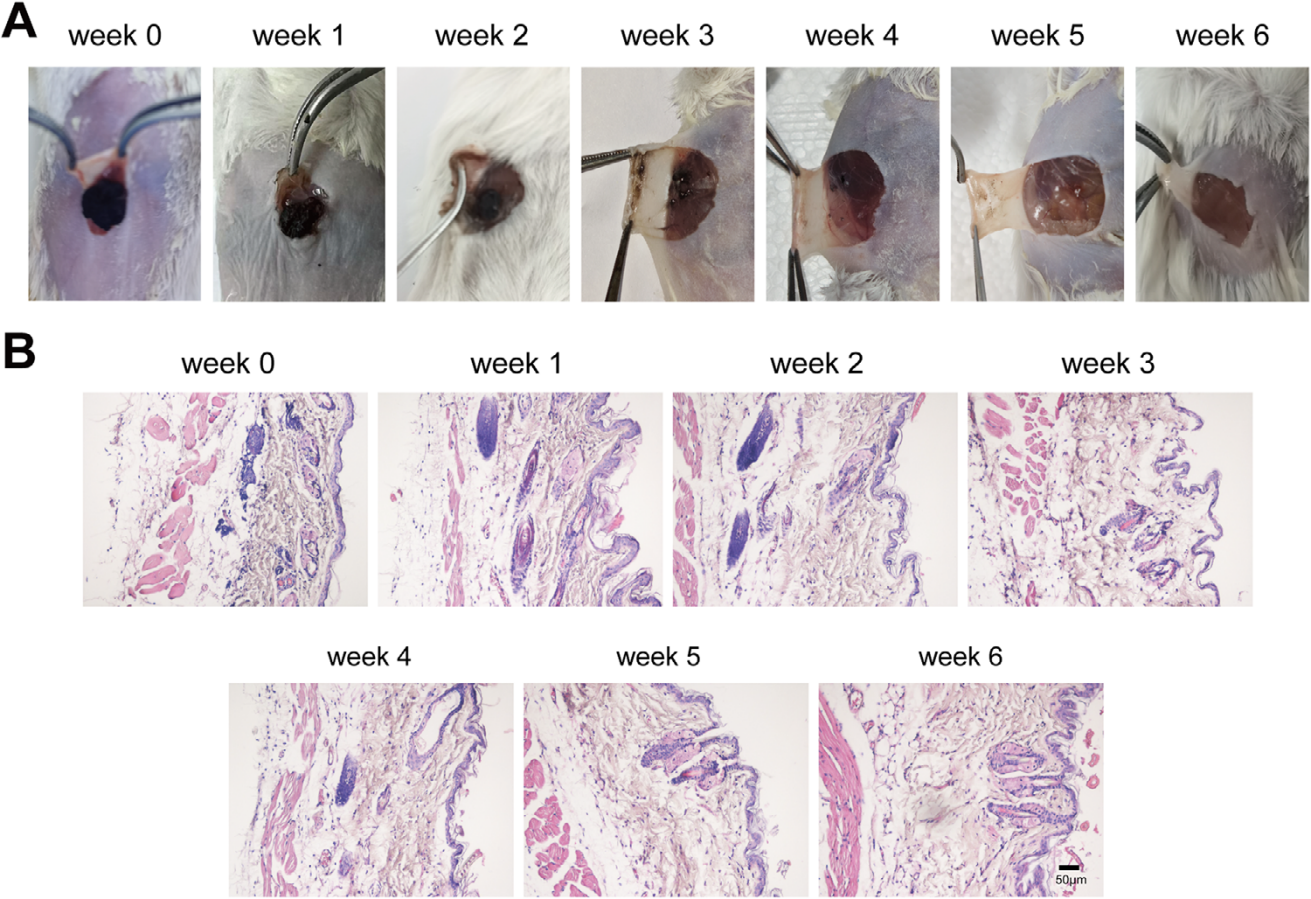
Subcutaneous degradation of the Gel/TA/Fe^3+^ in vivo. (A) In vivo subcutaneous degradation process of the Gel/TA/Fe^3^^+^ hydrogel. (B) H&E staining of the skin tissue. Scale bar = 50 µm.

### 3.6 Inhibition of Postoperative Tumor Recurrence of Gel/TA/Fe^3+^ In Vivo

Immune checkpoint inhibitors have been approved for the treatment of various cancers [37]. The anti‐tumor effect of the Gel/TA/Fe^3+^‐mediated mild PTT combined with αPD‐L1 on CRC was further investigated in vivo. A tumor‐bearing xenograft mice model was initially established by subcutaneously injecting CT26‐Luc cancer cells (2 × 10^6^ cells) into the right flank of Balb/c mice (Figure 6A). The mice were randomized into five groups: (1) saline control; (2) Gel/TA/Fe^3+^; (3) αPD‐L1; (4) Gel/TA/Fe^3+^ +L; and (5) Gel/TA/Fe^3+^ + L + αPD‐L1. On day 7, the subcutaneous tumors were surgically resected, leaving approximately 10% residual tumor to simulate postoperative model, and Gel/TA/Fe^3+^ was coated into the tumor resection cavity. As indicated in Figure S10A,B, the temperature of Gel/TA/Fe^3+^ + L group gradually rose to 45°C and then remained around 45°C during irradiation, which could not exert anytoxicity on the surrounding normal tissues. At the same time, we conducted 3 times of photothermal cycles at the tumor site of mice during treatment session of PTT to demonstrate the reproducibility of temperature control across different irradiation sessions. As shown in Figure S10C, the temperature of the Gel/TA/Fe^3+^ +L and Gel/TA/Fe^3+^ + L + αPD‐L1 groups remained at around 45°C after 5 min of irradiation, indicating that the Gel/TA/Fe^3+^ exhibits reproducible photothermal effects. The temperature of the adjacent normal tissues was around 37.5°C, significantly lower than the tumor temperature, indicating that the mild PTT did not cause thermal damage to the normal tissues (Figure S10D). As shown in Figure 6B–F, the Gel/TA/Fe^3+^ + L + αPD‐L1 group exhibited the strongest tumor‐suppressive efficacy, whereas the Gel/TA/Fe^3+^ group and αPD‐L1 monotherapy group showed limited tumor growth inhibition. This superior performance was attributed to the synergistic effects of Gel/TA/Fe^3+^‐mediated mild PTT and relieved immunosuppressive TME, which was achieved by inducing ferroptosis in tumor cells and M2‐type TAMs. Notably, the efficacy was further amplified by αPD‐L1, as indicated by the elimination of one out of six tumors. There was no significant change in body weight during the entire treatment (Figure 6C). The Gel/TA/Fe^3+^ + L + αPD‐L1 group also exhibited significantly extended survival time (more than 60 days), compared with other groups (Figure 6G). Moreover, the performance of Gel/TA/Fe^3+^ to induce GPX4 inactivation and GSSG accumulation in tumor tissues was evaluated. The most favorable GSSG generation was detected in Gel/TA/Fe^3+^ + L + αPD‐L1 treated tumor tissues (Figure 6H). The Western blotting analysis showed that Gel/TA/Fe^3+^ + L + αPD‐L1 caused the most pronounced downregulation of GPX4 expression in tumors (Figure 6I). These above two hallmark events of ferroptosis suggested that Gel/TA/Fe^3+^ + L + αPD‐L1 could induce ferroptosis in the tumor.

**FIGURE 6:**
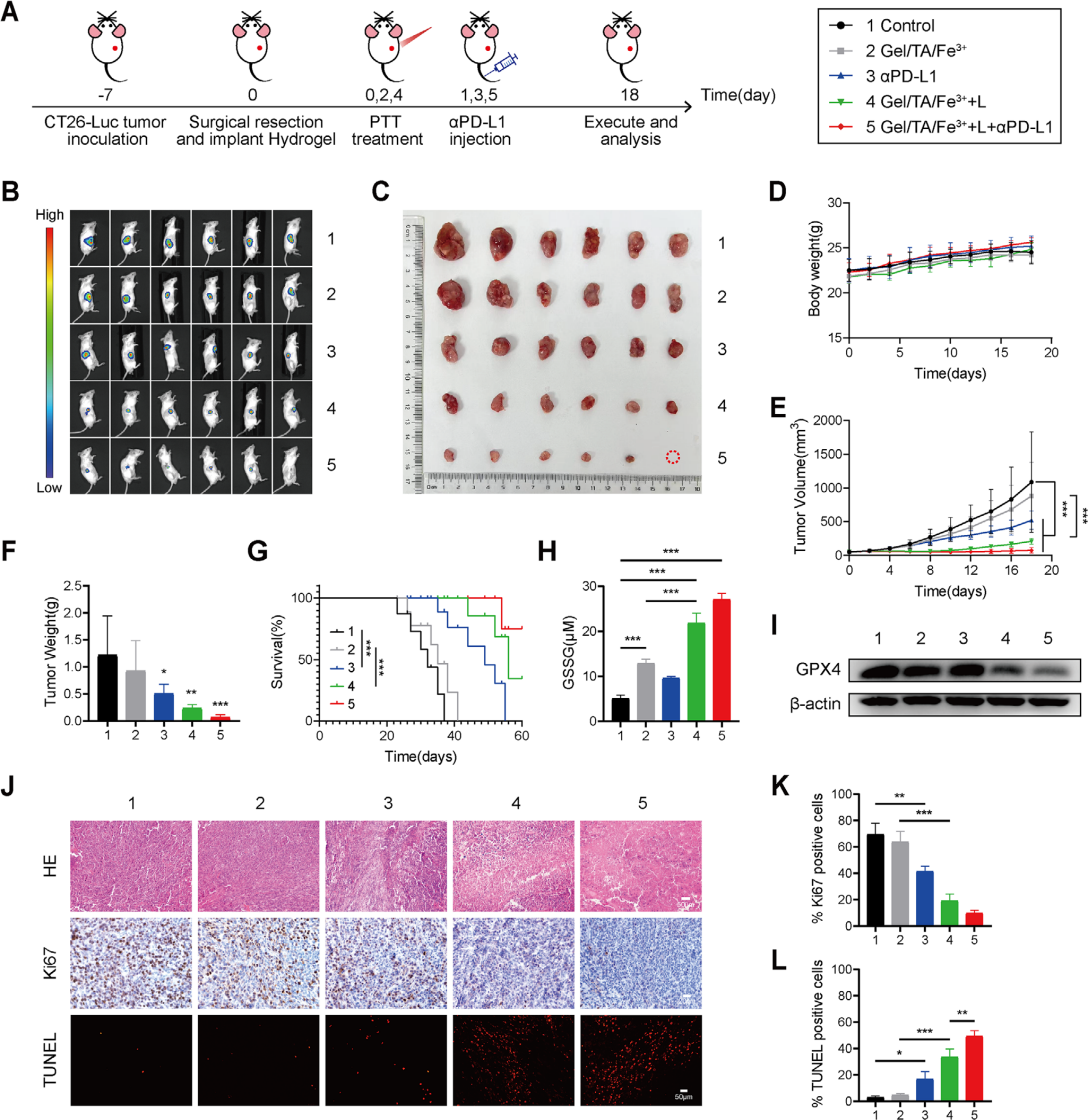
Inhibition of postoperative tumor recurrence of Gel/TA/Fe^3+^ in vivo. (A) Schematic diagram of the experimental timeline and therapeutic intervention (n = 6). (B) In vivo bioluminescence images of tumor‐bearing mice after different treatment. (C) Photographs of tumors from mice in different treatment groups at the study endpoint. (D) Body weight changes of mice throughout the treatment period. (E, F) Tumor growth curves and tumor weight. (G) Survival curves of mice (n = 6). (H) The GSSG concentration in tumor tissues from each group (mean ± SD; n = 3). (I) Western blot analysis of GPX4 protein expression in tumor tissues. (J) H&E (Scale bar = 50 µm), Ki67 (Scale bar = 20 µm), and TUNEL staining (Scale bar = 50 µm) of tumor tissues. (K, L) Quantification of Ki67‐positive and TUNEL positive cells (mean ± SD; n = 3). *p < 0.05, **p < 0.01 and ***p < 0.001.

In addition, the tumor tissues were harvested from the mice and stained for H&E, Ki‐67 and TUNEL staining (Figure 6J–L). The Gel/TA/Fe^3+^ + L + αPD‐L1 group showed the most extensive necrosis and apoptosis and the lowest proliferation index, demonstrating that Gel/TA/Fe^3+^ ‐mediated mild PTT and ferroptosis combined with αPD‐L1 effectively suppressed tumor growth. Tumor tissue sections were also subjected to CD4, CD8, and GZMB immunofluorescence staining (Figure S11). The Gel/TA/Fe^3+^ + L + αPD‐L1 group exhibited the strongest green fluorescence, indicating that the combination therapy enhanced the infiltration of CD4^+^ and CD8^+^ T cells in tumor site. Furthermore, the highest GZMB green fluorescence intensity was observed in the Gel/TA/Fe^3+^ + L + αPD‐L1 group, suggesting that this combination therapy promotes the cytotoxic function of CD8^+^ T cells within the TME. Detection of cytokine levels in serum revealed that pro‐inflammatory cytokines associated with DC maturation (IL‐6, TNF‐α, IFN‐γ, IL‐12p70) were upregulated in the Gel/TA/Fe^3+^ + L and Gel/TA/Fe^3+^ + L + αPD‐L1 groups, while the M2‐associated anti‐inflammatory cytokine IL‐10 was downregulated (Figure S12). Our findings indicated that the combination of Gel/TA/Fe^3+^‐mediated mild PTT and αPD‐L1 can activate anti‐tumor immune response probably by inducing DC maturation and reduction in M2 macrophages.

### 3.7 Activation of Anti‐Tumor Immunity In Vivo

To elucidate the underlying mechanisms of the anti‐tumor efficacy, immune cells in the lymph nodes, spleens, and tumor tissues from each group were harvested and analyzed by flow cytometry. First, we investigated DCs in the lymph nodes, which play vital roles in activating adaptive anti‐tumor immunity. As shown in Figure 7A, the Gel/TA/Fe^3+^ + L (5.77‐fold) and Gel/TA/Fe^3+^ + L + αPD‐L1 (9.34‐fold) groups effectively promoted DC maturation compared with the control group. Next, an increased population of CD3^+^CD8^+^ T and CD3^+^CD4^+^ T cells was observed in splenic tissues, with the most prominent elevation in mice treated with Gel/TA/Fe^3+^ + L + αPD‐L1 among all groups. This finding indicated that Gel/TA/Fe^3+^ under laser irradiation combined with αPD‐L1 triggers a T cell‐mediated adaptive anti‐tumor immune response (Figure 7B). Effector memory T cells (Tem, defined as CD3^+^CD8^+^CD44^+^CD62L^−^) in the spleens of mice were further detected by flow cytometry. The Gel/TA/Fe^3+^ + L + αPD‐L1 group generated the highest number of Tem cells (Figure 7C), demonstrating that the combination treatment induces an immune memory response. As illustrated in Figure 7D, the number of natural killer (NK) cells in the spleen was significantly increased in the Gel/TA/Fe^3+^ + L + αPD‐L1 group, suggesting that PTT promotes innate immunity. In addition, myeloid‐derived suppressor cells (MDSCs) are known to inhibit T cell proliferation and function by downregulating the expression of interleukin‐2 (IL‐2), interferon‐γ (IFN‐γ), and granzyme B [38]. MDSCs also reduce the number of CD8^+^ T cells and impair their cytotoxicity against tumor cells. As shown in Figure 7E, the number of MDSCs was notably decreased following treatment with Gel/TA/Fe^3+^ + L + αPD‐L1, indicating alleviation of the immunosuppressive TME. Meanwhile, M1‐type TAMs were increased, whereas M2‐type TAMs were decreased in the Gel/TA/Fe^3^^+^ + L and Gel/TA/Fe^3+^ + L + αPD‐L1 groups, which is consistent with our in vitro findings (Figure 7F,G). Collectively, these data demonstrated that Gel/TA/Fe^3+^‐mediated mild PTT and ferroptosis combined with αPD‐L1 effectively reverse the immunosuppressive TME in vivo, converting “cold tumors” into “hot tumors” and thereby activating systemic immunity to eradicate tumor cells.

**FIGURE 7:**
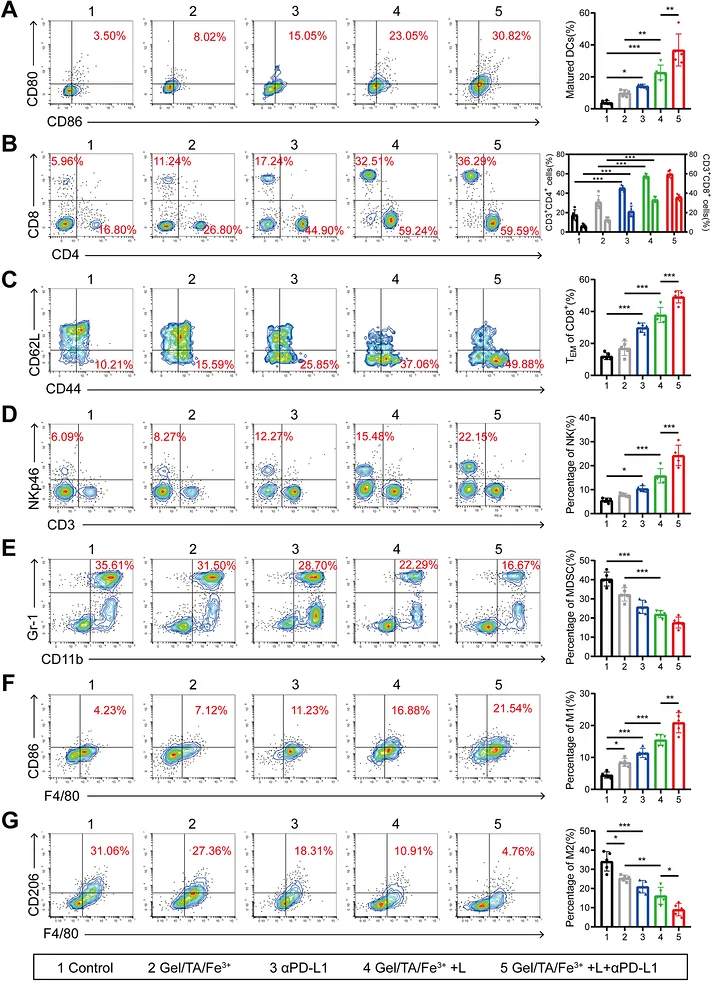
Flow cytometry analysis of activation of anti‐tumor immunity in vivo. (A) Proportions of mature DCs (CD11c^+^CD80^+^CD86^+^) in lymph node. (B) Proportions of CD3^+^CD8^+^, CD3^+^CD4^+^ T cells in spleen. (C) Proportions of Tem cells (CD3^+^CD8^+^CD44^+^CD62L^−^) in spleen. (D) Proportions of NK cells (CD3^−^NKp46^+^) in spleen. (E) Proportions of MDSCs (CD11b^+^Gr‐1^+^) in spleen. (F) Proportions of M1‐type TAMs (CD11b^+^F4/80^+^CD86^+^) in tumor. (G) Proportions of M2‐type TAMs (CD11b^+^F4/80^+^CD206^+^) in tumor. Mean ± SD; n = 5. *p < 0.05, **p < 0.01 and ***p < 0.001.

### 3.8 Inhibition of Tumor Metastasis of Gel/TA/Fe^3+^ In vivo

To investigate the anti‐metastatic efficacy of Gel/TA/Fe^3+^‐mediated mild PTT in combination with αPD‐L1, a mice lung metastatic tumor model was established, as tumor metastasis remains the primary contributor to the poor prognosis of CRC patients. As illustrated in Figure 8A, 2 × 10^6^ CT26 cells were subcutaneously injected into the right flank of mice to establish primary tumors, then 1 × 10^6^ CT26 cells were administered via tail vein injection to induce lung metastases. As shown in Figure 8B–E and Figure S13, the Gel/TA/Fe^3+^ + L and Gel/TA/Fe^3+^ + L + αPD‐L1 groups exhibited significant suppression of lung metastasis, characterized by a marked reduction in the number of pulmonary nodules compared to the control groups. Notably, no significant fluctuations in body weight were observed in mice throughout the treatment period (Figure 8C), confirming the favorable biosafety profile of the Gel/TA/Fe^3^^+^ hydrogel. Furthermore, the Gel/TA/Fe^3+^ + L + αPD‐L1 combination group demonstrated a substantial extension of overall survival compared to all other treatment groups (Figure 8F), highlighting the synergistic therapeutic benefit of mild PTT and immune checkpoint blockade. Histopathological examination via H&E staining of lung sections further validated that the Gel/TA/Fe^3+^ + L + αPD‐L1 group had the lowest density of metastatic foci (Figure 8G). Immunofluorescence staining for CD8 and GZMB in lung tissues revealed the strongest green fluorescence signal in the Gel/TA/Fe^3+^ + L + αPD‐L1 group (Figure 8H and Figure S14), indicating enhanced infiltration of effector CD8^+^ T cells into metastatic lesions. Consistently, this group also exhibited the highest GZMB expression, suggesting increased cytotoxic potential of tumor‐infiltrating CD8^+^ T cells. Collectively, these findings demonstrated that the potent anti‐metastatic activity of Gel/TA/Fe^3+^‐mediated mild PTT in CRC is attributed to the synergistic induction of ICD and ferroptosis by the hydrogel, thereby potentiating the efficacy of immunotherapy against metastatic tumors.

**FIGURE 8:**
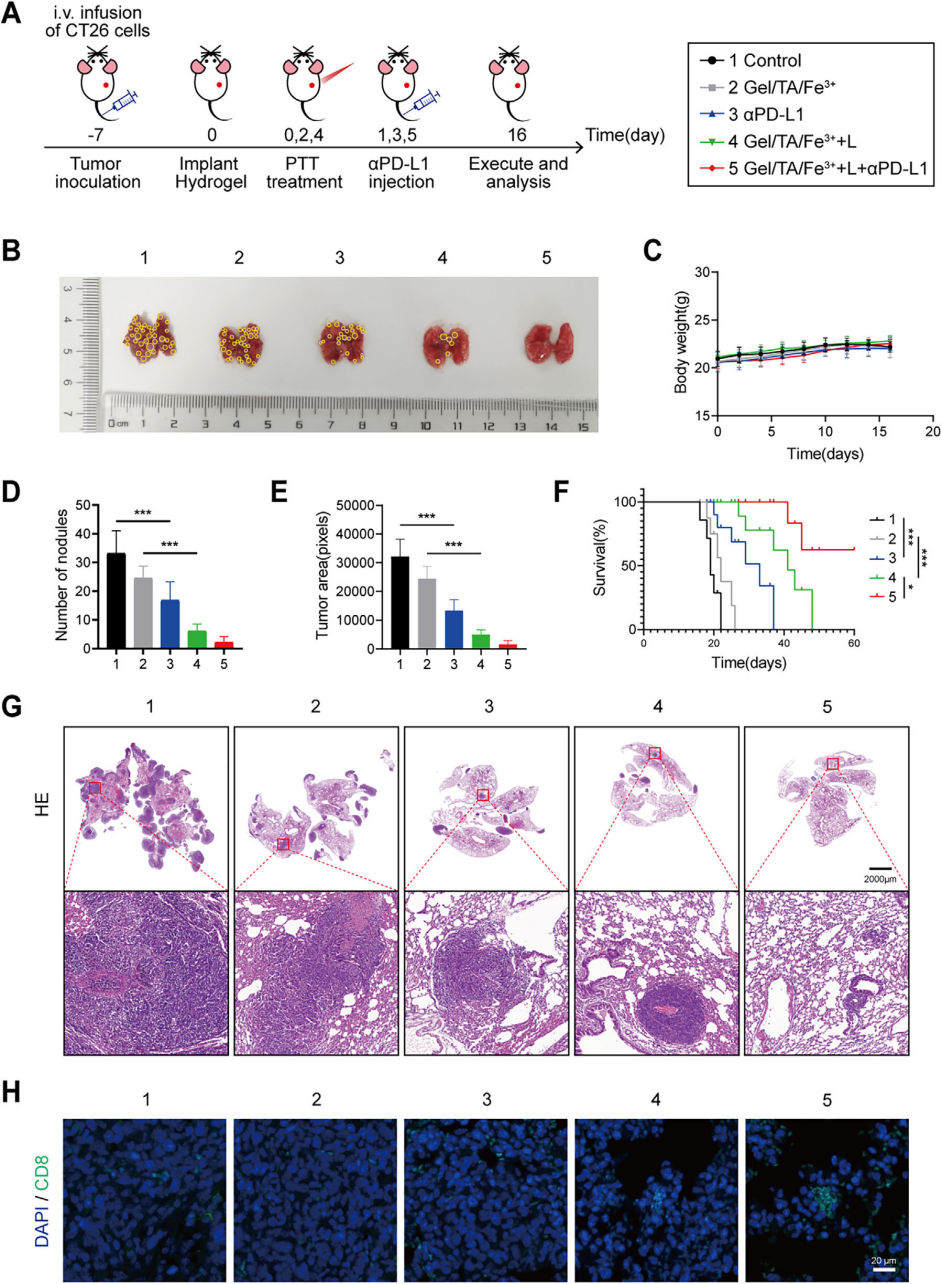
Inhibition of lung metastasis of Gel/TA/Fe^3+^ in vivo. (A) Schematic diagram of the experimental timeline and therapeutic intervention (n = 6). (B) Representative Lung images with metastatic nodules from each treatment group. (C) Body weight changes of mice throughout the treatment period. (D) The number of surface metastatic nodules in the lungs of each group (mean ± SD; n = 6). (E) Total tumor area in the lung sections (mean ± SD; n = 6). (F) Survival curves of mice (n = 6). (G) H&E staining of lung tissues. Scale bar = 2000 µm. (H) Immunofluorescence images of CD8 in the lung sections (CD8, green fluorescence; DAPI, blue fluorescence). Scale bar = 20 µm. *p < 0.05 and ***p < 0.001.

### 3.9 Biosafety Evaluation of Gel/TA/Fe^3+^

To evaluate the in vivo biosafety of the Gel/TA/Fe^3+^, a systematic assessment was performed including histopathological examination and serum biochemical analysis. First, major organs (heart, liver, spleen, lungs, and kidneys) were harvested from mice, followed by H&E staining for histopathological observation. As illustrated in Figure 9A, no obvious pathological abnormalities (e.g., tissue necrosis, edema, inflammatory cell infiltration, or structural destruction) were detected in any of the examined organs across all treatment groups. Furthermore, key biochemical markers in serum were analyzed, including alanine transaminase (ALT), aspartate transaminase (AST), creatinine (CRE), and blood urea nitrogen (BUN) (indicators of renal function). As shown in Figure 9B, the levels of ALT, AST, CRE, and BUN in all treatment groups remained within the normal physiological ranges, with no statistically significant differences compared to the control group. Collectively, these results demonstrated that the Gel/TA/Fe^3+^ exhibits excellent in vivo biosafety.

**FIGURE 9:**
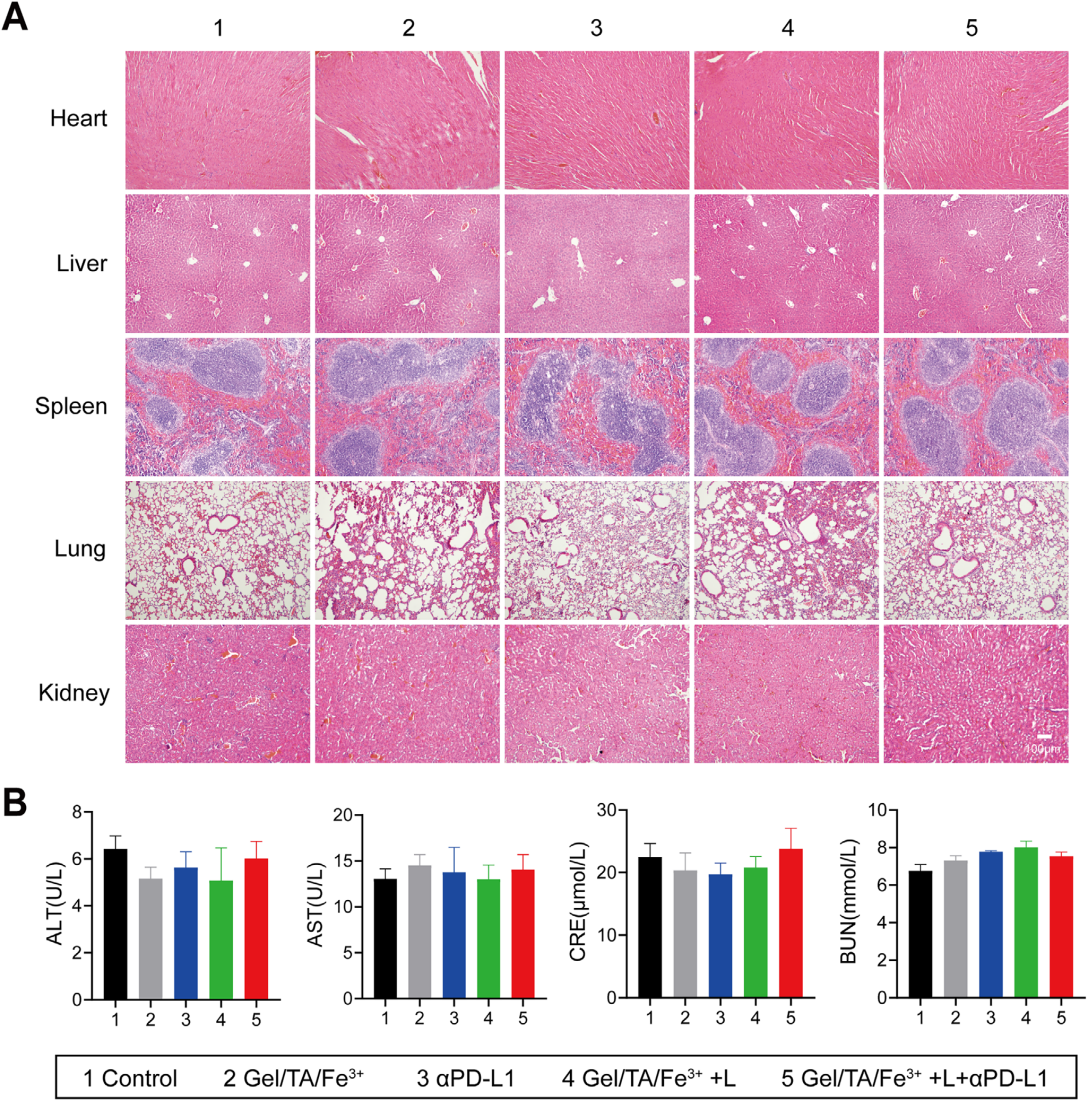
Biosafety evaluation. (A) H&E staining of major organs (heart, liver, spleen, lung, and kidney). Scale bar = 100 µm. (B) Biochemical analysis results of ALT, AST, BUN, and CRE (mean ± SD; n = 3).

## 4 Discussion

The high mortality of CRC is closely associated with postoperative local recurrence and distant metastasis. Additionally, an immunosuppressive TME renders immune checkpoint inhibitors ineffective for most CRC patients. How to reshape the immunosuppressive TME, eliminate residual tumor cells after surgery, and inhibit recurrence and metastasis remains a major challenge in clinical practice. To address this issue, we designed an injectable iron‐tannic acid hydrogel for locally mild PTT (≤ 45°C) in combination with immunotherapy to prevent the postoperative recurrence and metastasis of CRC. Through the strategy of PTT‐induced ICD and dual ferroptosis, a robust anti‐tumor immune response was activated, and in combination with αPD‐L1, the postoperative recurrence and metastasis of CRC were effectively inhibited.

The Gel/TA/Fe^3+^ system is primarily composed of gelatin, TA, and iron(III) chloride (FeCl_3_). Gelatin is derived from collagen hydrolysis, TA is a plant‐derived polyphenol, and Fe^3+^ is an essential trace element in the human body. Since all components are either natural or endogenous, the Gel/TA/Fe^3+^ system exhibits excellent biocompatibility and biosafety, significantly reducing potential toxic side effects on tissues. In contrast to the direct assembly of gelatin with TA, which lacks both injectability and adhesiveness, the assembly of gelatin microspheres with TA achieves both properties. Furthermore, TA chelates with Fe^3+^, enhancing photothermal conversion efficiency and inducing ferroptosis. Gel/TA/Fe^3+^ offered multiple benefits. These favourable characteristics enable it to be injected into irregular postoperative wound beds and cavities, as well as firm adhesion to tissue surfaces. Subsequently, the Gel/TA/Fe^3+^ hydrogel system is irradiated, with the local temperature maintained below 45°C. The hydrogel‐mediated mild PTT not only induces tumor cell apoptosis but also triggers ICD, thereby promoting the maturation of DCs and further initiating T cell‐mediated immune responses. Compared with traditional high‐temperature PTT (>50°C), mild PTT avoids damage to normal tissues and reduces the thermal tolerance caused by the production of heat shock proteins (HSPs) [39]. Simultaneously, the Gel/TA/Fe^3+^ system can induce ferroptosis both in tumor cells and TAMs through Fenton reactions within the TME. Critically, M2‐type TAMs are more susceptible to ferroptosis than M1‐type TAMs. Ferroptosis induced by Gel/TA/Fe^3+^ enables the selective depletion of M2‐type TAMs, thereby alleviating the immunosuppressive TME. Through the synergistic effects of tumor cell ICD and dual ferroptosis, Gel/TA/Fe^3+^ mediated mild PTT could effectively converts a “cold” TME into an immunologically active “hot” TME, thus the anti‐tumor efficacy achieved by Gel/TA/Fe^3+^‐mediated mild PTT is nearly comparable to that of hyperthermic PTT. Once further combined with αPD‐L1, the system reverses T cell‐mediated immunosuppression and activates systemic anti‐tumor immune responses in vivo. Collectively, these results demonstrate that Gel/TA/Fe^3+^‐mediated mild photothermal therapy potentiates the anti‐tumor efficacy of αPD‐L1, achieving effective suppression of postoperative CRC recurrence and metastasis.

We have successfully developed a multifunctional Gel/TA/Fe^3+^ hydrogel, and this system mediated mild PTT can effectively inhibit postoperative recurrence and metastasis of CRC. The sublethal or lethal effects of mild photothermal therapy could kill tumor cells with high metabolic activity and heat sensitivity, while sparing normal cells and surrounding tissues. Meanwhile, acting locally on the tumor tissues could avoid the potential collateral tissue damage caused by high temperature. Unlike conventional anti‐tumor modalities with widespread toxicity or destructiveness, mild PTT acts in a localized and targeted manner. Therefore, its low invasive performance, low treatment intensity, good biosafety, adjustable irradiation conditions, and excellent repeatability make it more acceptable for clinical transformation and long‐term in vivo application.

Despite the promising therapeutic outcomes in murine CRC models, several important challenges remain to be addressed for future clinical translation of this hydrogel system. First, regarding compatibility with standard human colorectal cancer surgery, the injectable and in situ gelation characteristics of the Gel/TA/Fe^3+^ system are favorable for minimally invasive or open surgical procedures, allowing precise deposition onto the post‐resection tumor bed without disrupting conventional anastomosis, hemostasis, or irrigation steps. Further optimization of gelation kinetics and mechanical strength will be required to ensure reliable performance during intraoperative manipulation and tissue handling. Second, scalable and reproducible fabrication under GMP conditions is achievable given the simple, solvent‐free mixing preparation using commercially available pharmaceutical‐grade tannic acid and iron precursors. Future scale‐up efforts will focus on standardized formulation protocols, real‐time monitoring of crosslinking efficiency, and consistent batch‐to‐batch quality control. Third, for clinical sterilization, terminal high‐temperature or high‐dose radiation methods may compromise the dynamic TA‐Fe coordination network and adhesive properties; thus, we propose sterile filtration of aqueous precursor solutions followed by bedside mixing as a practical strategy, with low‐temperature electron‐beam irradiation as an alternative terminal sterilization approach for pre‐assembled hydrogels. Fourth, in clinically relevant scenarios involving surgical bleeding and interstitial tissue fluid exudation, the strong wet‐tissue adhesion and hemostatic capability of tannic acid‐based networks help maintain gel integrity and therapeutic function in bloody, exudative microenvironments. Although limited NIR light penetration remains a constraint, the use of interventional fiber optics and endoscopic irradiation for topical application directly to the residual tumor bed ensures sufficient energy deposition to the high‐risk microscopic residual disease region, thereby maximizing local therapeutic efficacy [40]. Fifth, while the current study relies on systemic αPD‐L1 administration to amplify local photothermal‐induced immunity, future refinement of immune checkpoint delivery could further enhance therapeutic index. Inspired by Liu et al., who utilized intraperitoneally injected ROS‐responsive hydrogels for local delivery of immune checkpoint antibodies, future nanoparticle encapsulation of αPD‐L1 could enable on‐demand and controlled release of αPD‐L1 [41]. Such a strategy would allow local retention at the postoperative cavity while still permitting a fraction of the antibody to enter systemic circulation for micrometastasis surveillance, potentially reducing immune‐related adverse events and dosing frequency. Collectively, these translational considerations highlight both the practical potential and necessary improvements required to advance this combinatorial photothermal—ferroptosis immunotherapy toward clinical application in postoperative colorectal cancer.

## 5 Conclusions

In summary, we demonstrated a strategy of combining mild photothermal therapy with ferroptosis to enhance anti‐tumor immunity by spreading granular hydrogel (Gel/TA/Fe^3+^) to the wound site after tumor resection to inhibit postoperative recurrence and metastasis of CRC. Based on NHS/EDC‐crosslinked gelatin microspheres, assembled via hydrogen bonding with TA, it confers injectability and strong adhesiveness; further TA chelated with Fe^3+^ achieves efficient photothermal conversion. Gel/TA/Fe^3+^ functions as a photothermal agent to ablate tumor cells under NIR irradiation, inducing ICD and promoting DC maturation to initiate immune responses. Concurrently, Gel/TA/Fe^3+^ induces ferroptosis in colorectal cancer cells and M2‐type TAMs through Fenton reactions, thereby effectively reversing the immunosuppressive TME. In synergy with αPD‐L1 immune checkpoint blockade, this approach robustly suppresses postoperative CRC recurrence and distant pulmonary metastasis. These findings provide an efficient and translational strategy for the treatment of advanced CRC.

## Author Contributions

**Yuan Zhu**: methodology, software, validation, writing – original draft, writing – review and editing, data curation. **Chang Lu**: validation. **Peihao Yin**: supervision, funding acquisition, project administration. **Wanyi Liu**: software, data curation. **Tao Wang**: software, data curation. **Xi Yu**: writing – original draft, writing – review and editing, investigation, formal analysis. **Jiani Zhong**: validation. **Yujie He**: validation. **Zeting Yuan**: writing – review and editing, conceptualization, supervision, funding acquisition, project administration. **Kunxi Zhang**: conceptualization, writing – original draft, resources. **Wei Li**: methodology.

## Conflicts of Interest

The authors declare no conflicts of interest.

## Supporting information




**Supporting File**: advs77186‐sup‐0001‐SuppMat.docx.

## Data Availability

All data are available in the main text and supporting information, and are also available upon request from the corresponding author.
