# Supplementary material for: Iron‐Tannic Acid Hydrogel Mediated Mild Photothermal Therapy Combined With Ferroptosis Enhances Anti‐Tumor Immunity in Colorectal Cancer
Source: Adv Sci (Weinh). 2026 Aug 25;13(63):e77186. doi: 10.1002/advs.77186 (PMC13505463; doi:10.1002/advs.77186)
Supplement: Supplementary file 1 [file ADVS-13-e77186-s001.docx]

**Supporting information**

**Figure S1.** (A) Macrograph of Gel and TA system. (B) Stability of Gel and TA system in deionized water for 48 h. (C) Observation of the extrusion properties of Gel and TA system.

**Figure S2.** Stability of Gel/TA/Fe^3+^ granular gel in PBS with 10% FBS and collagenase IV for 48 h.

**Figure S3.** The temperature rise curve of Gel/TA(10%)/Fe^3+^(0.1%) under NIR irradiation.

**Figure S4.** The temperature of hydrogels with different combinations of TA and Fe^3+^ concentrations under 5 min of NIR irradiation.

**Figure S5.** Fluorescence images of Calcein-AM/PI double staining of CT26 cells after treatment with Gel/TA/Fe^3+^. (The grouping was described in Materials and Methods 2.8.)

**Figure S6.** Cumulative release curves of Fe ions from Gel/TA/Fe^3+^ in PBS at pH 7.4 and pH 5.5. (n=3)

**Figure S7.** The colour change between Fe^3+^ and Fe^2+^ with o-phenanthroline.

**Figure S8.** Western blot analysis of ACSL4 protein expression in CT26 cells after treatment.

**Figure S9.** Flow cytometry analysis of iNOS expression in different types of macrophages and quantitative analysis (mean ± SD; n = 3). ***P < 0.001.

**Figure S10.** (A) Photothermal imaging of mice during 5 min of NIR irradiation (0–5 min) and (B) corresponding temperature changes at tumor sites. (C) Photothermal imaging of mice after 3 times of photothermal cycles of mice during treatment session of PTT and corresponding temperature at tumor sites. (D) Temperatures of the tumor and adjacent normal tissues after 5 min of NIR irradiation.

**Figure S11.** Immunofluorescence images of CD4, CD8 and GZMB in tumor tissues (CD4, CD8 and GZMB, green fluorescence; DAPI, blue fluorescence). Scale bar = 50μm.

**Figure S12.** Serum levels of inflammatory cytokines of IL-6, TNF-α, IFN-γ, IL-12p70, and IL-10 in mice (mean ± SD; n = 3). *P < 0.05, **P < 0.01 and ***P < 0.001.

**Figure S13.** Lung images with metastatic nodules from each treatment group (n=6).

**Figure S14.** Immunofluorescence images of GZMB in lung metastasis tissues (GZMB, green fluorescence; DAPI, blue fluorescence). Scale bar = 50μm.
